# Supplementary material for: Synthesis and Fluorescent Properties of Aminopyridines and the Application in “Click and Probing”
Source: Molecules. 2022 Feb 28;27(5):1596. doi: 10.3390/molecules27051596 (PMC8912075; doi:10.3390/molecules27051596)

^{1}H and ^{13}C NMR spectra

Figure S1. Diethyl 2-(*tert*-butylamino)-6-phenylpyridine-3,4-dicarboxylate (**1**, $CDCl_3$)

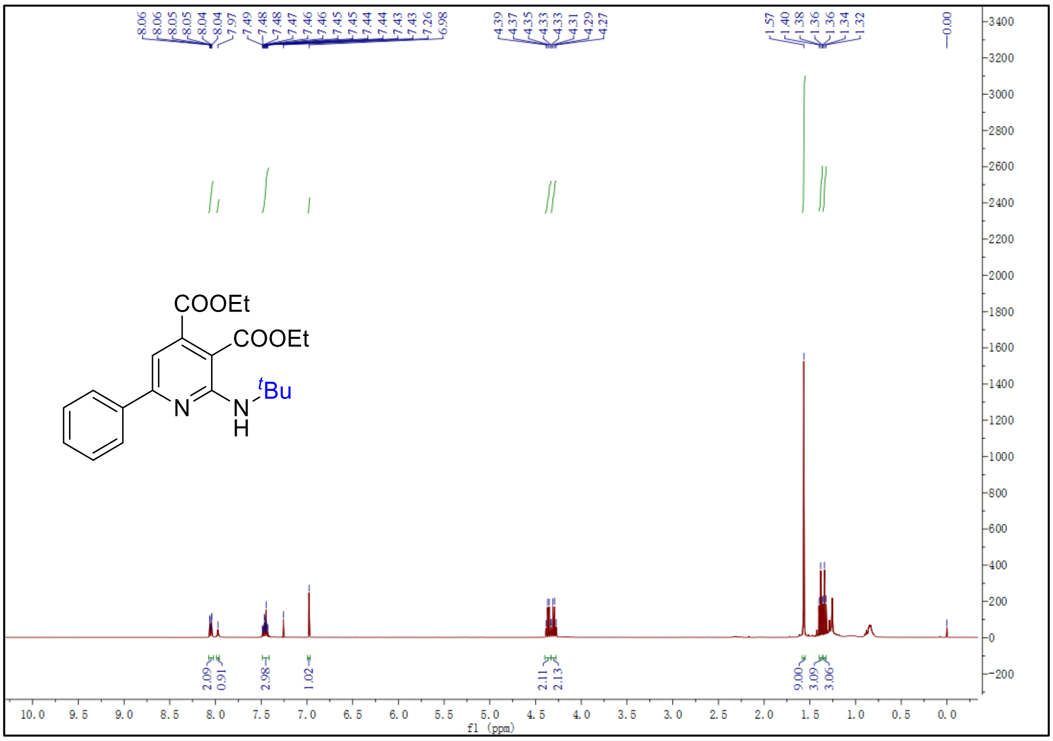

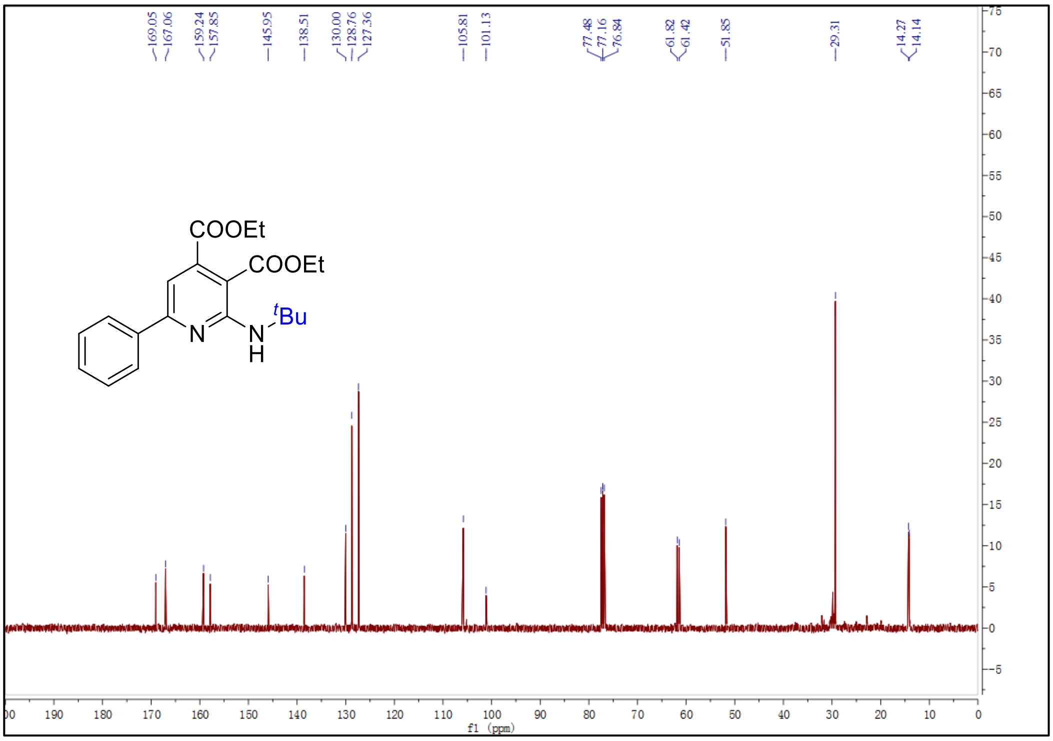

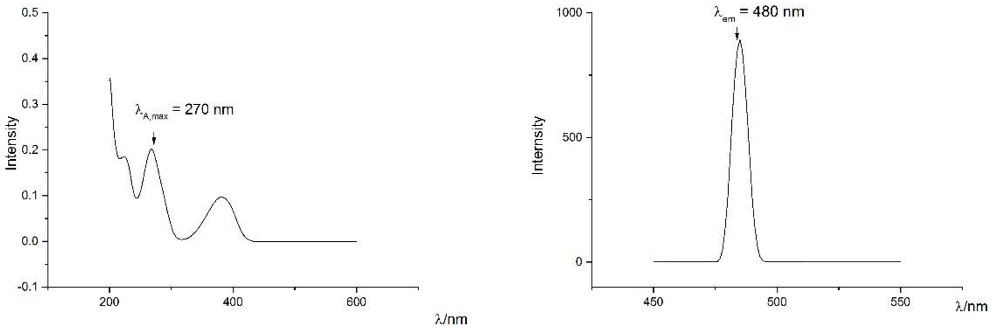

Figure S2. Diethyl 2-(benzylamino)-6-phenylpyridine-3,4-dicarboxylate (**2**, $CDCl_3$)

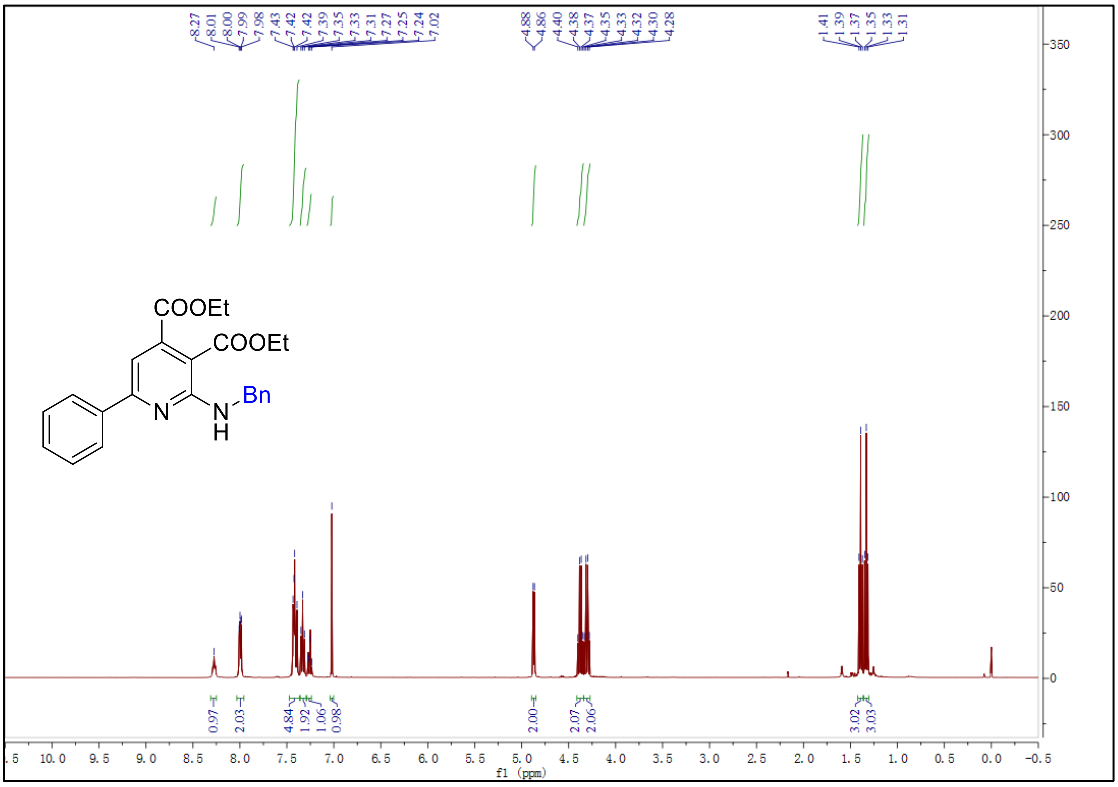

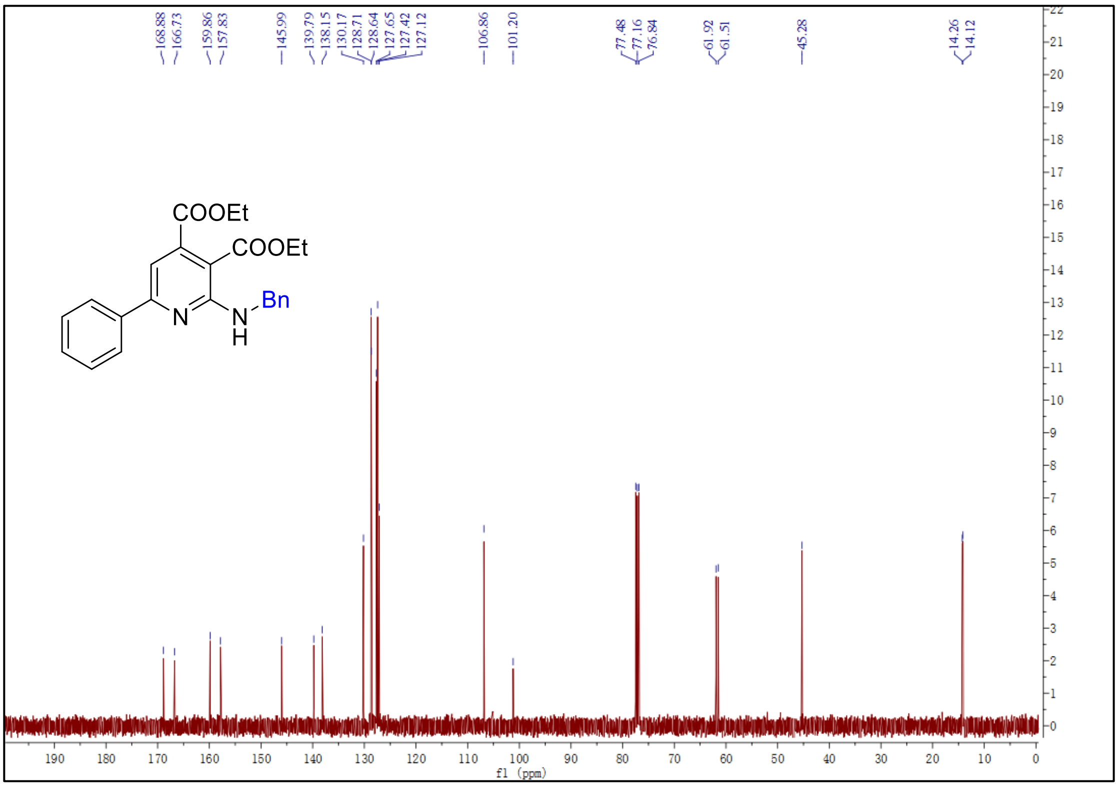

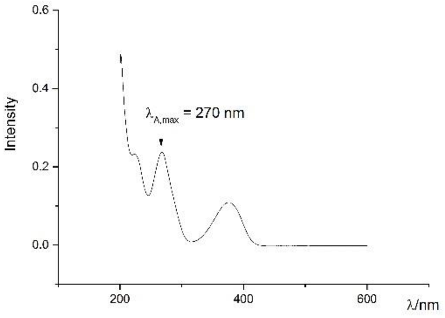

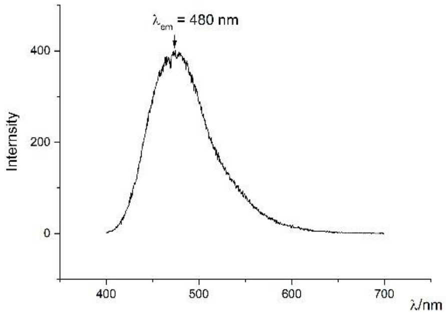

Figure S3. Diethyl 2-(cyclohexylamino)-6-phenylpyridine-3,4-dicarboxylate (**3**, $CDCl_3$)

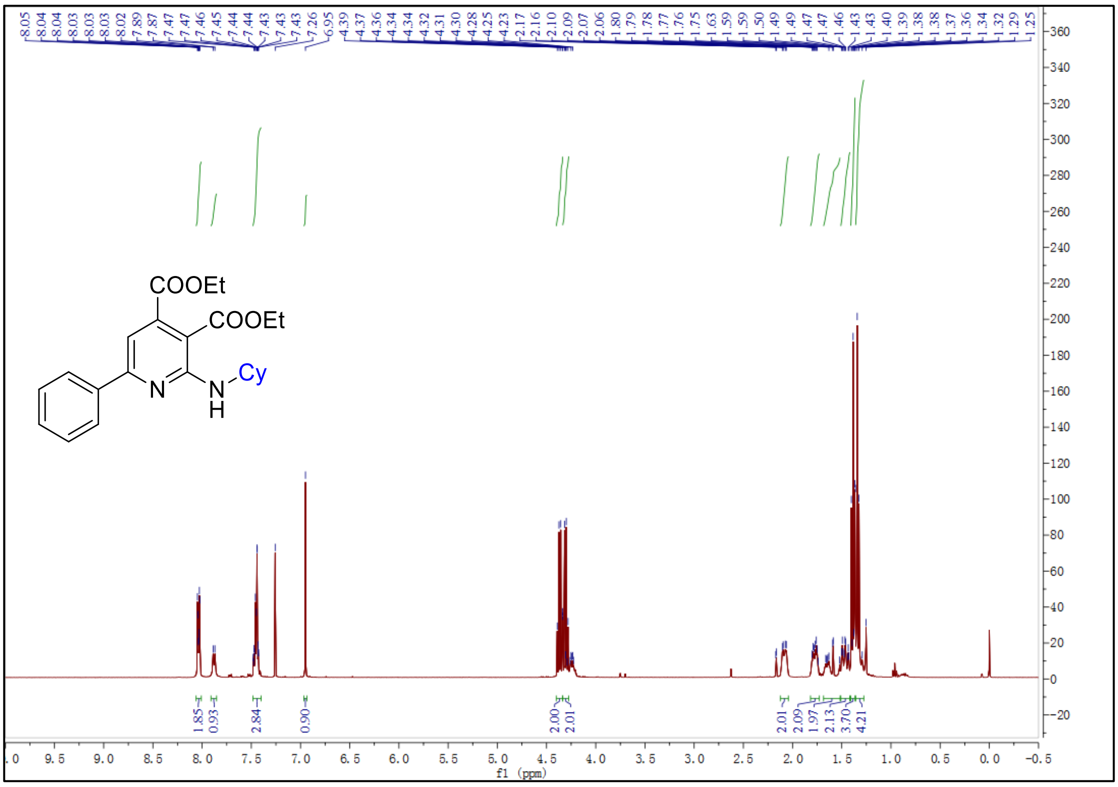

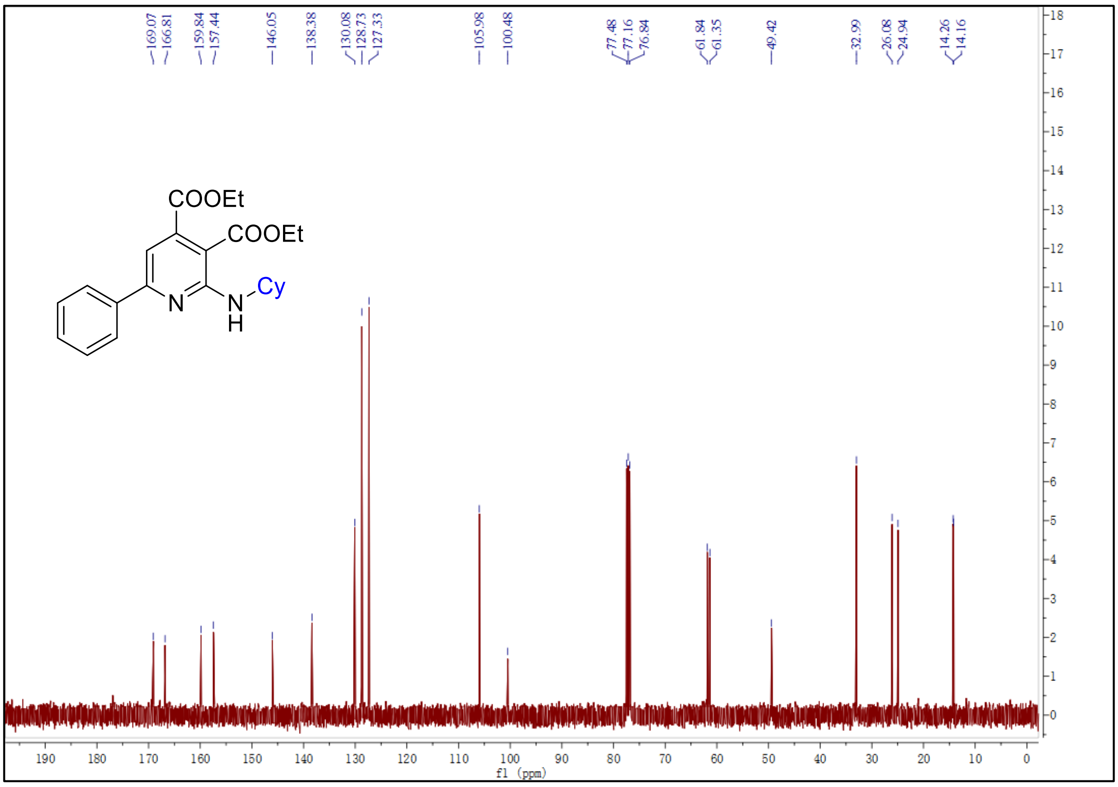

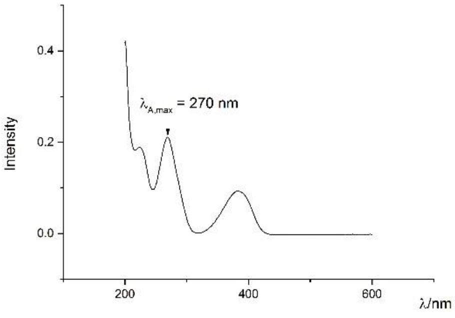

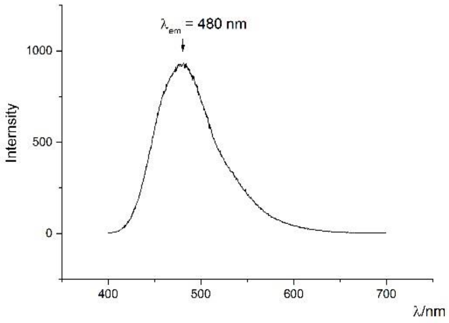

Figure S4. Diethyl 2-(*tert*-butylamino)-6-(4-(trifluoromethyl)phenyl)pyridine-3,4-dicarboxylate (**4**, $CDCl_3$)

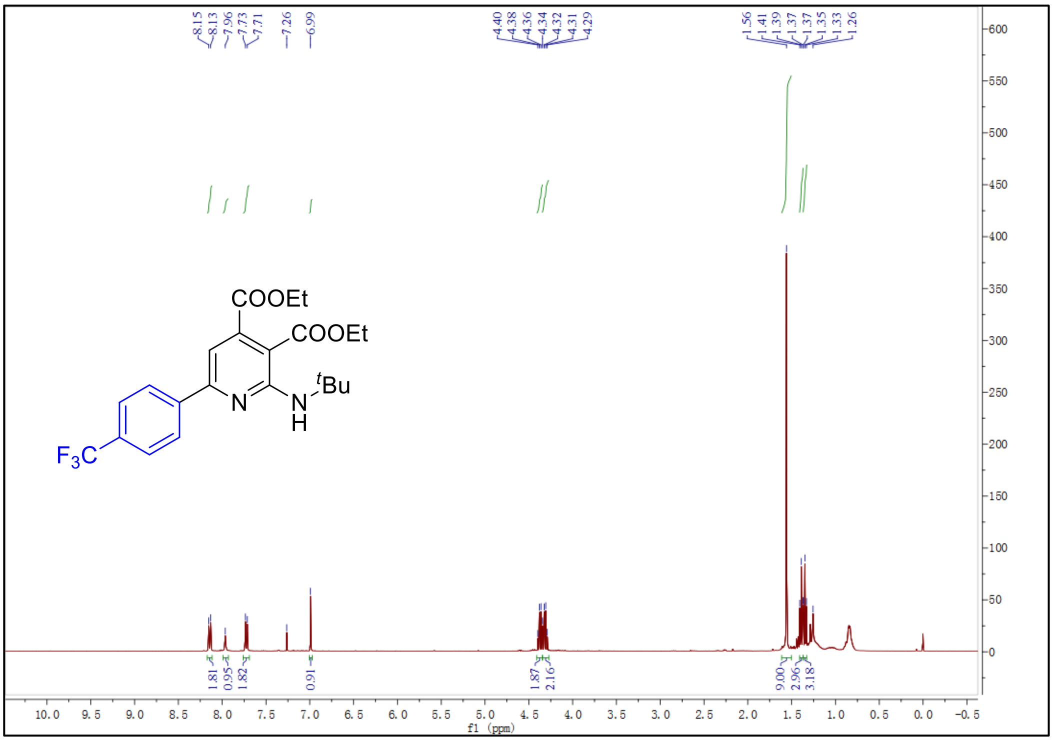

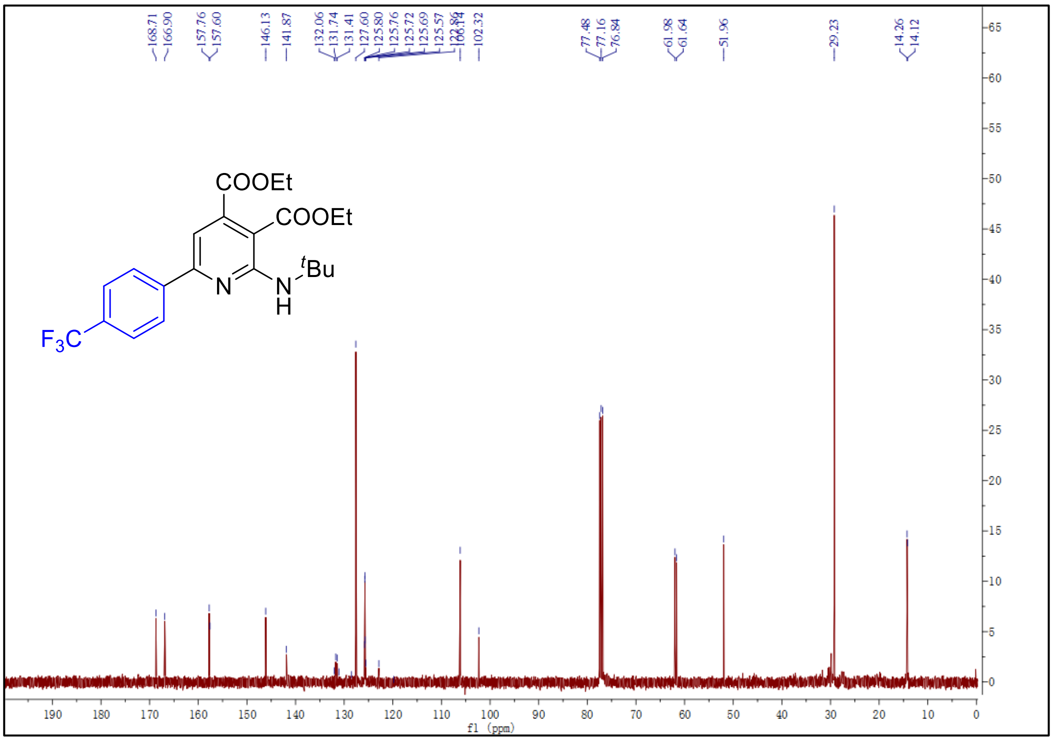

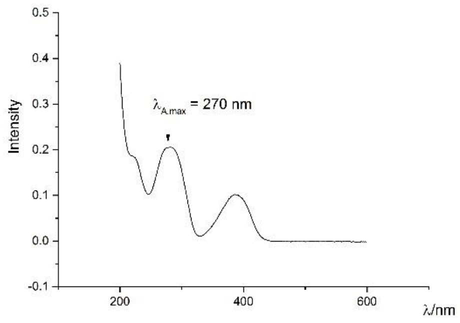

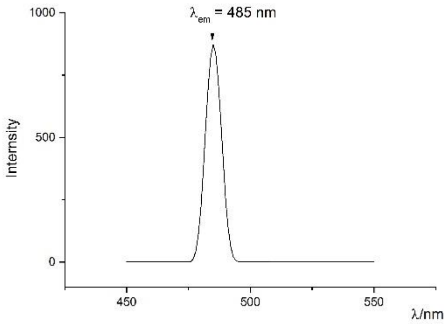

Figure S5. Diethyl 2-(*tert*-butylamino)-6-(p-tolyl)pyridine-3,4-dicarboxylate (**5**, $CDCl_3$)

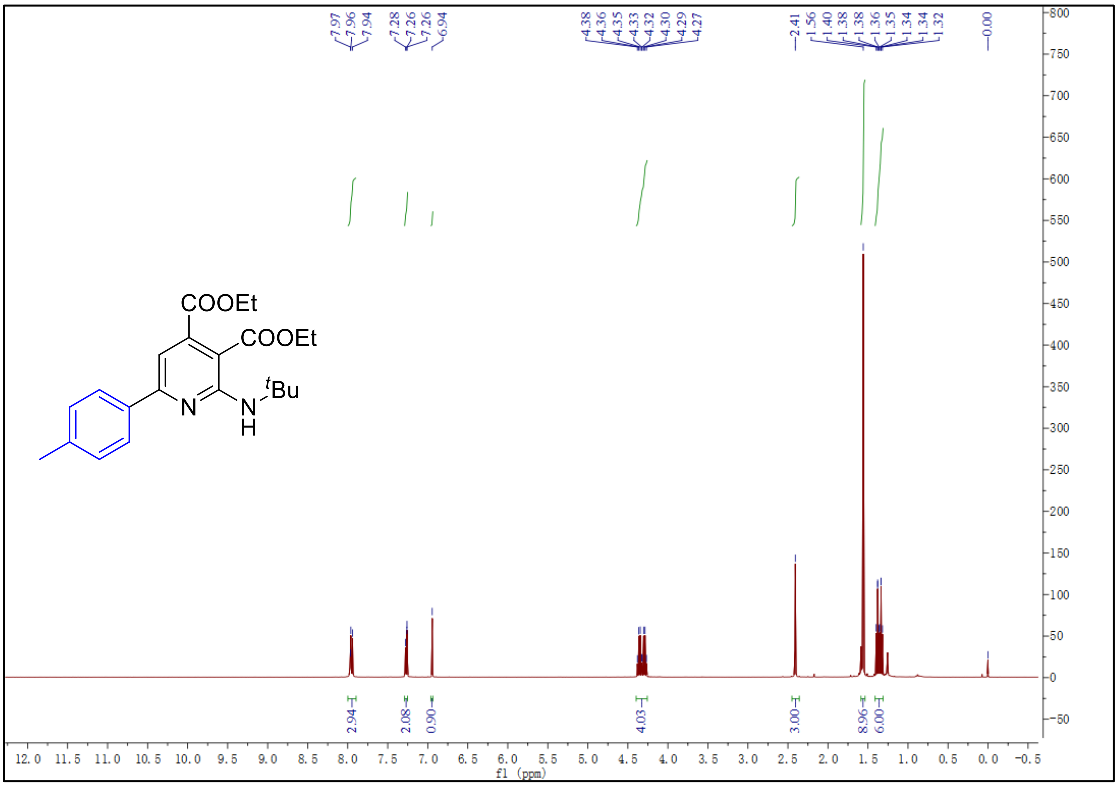

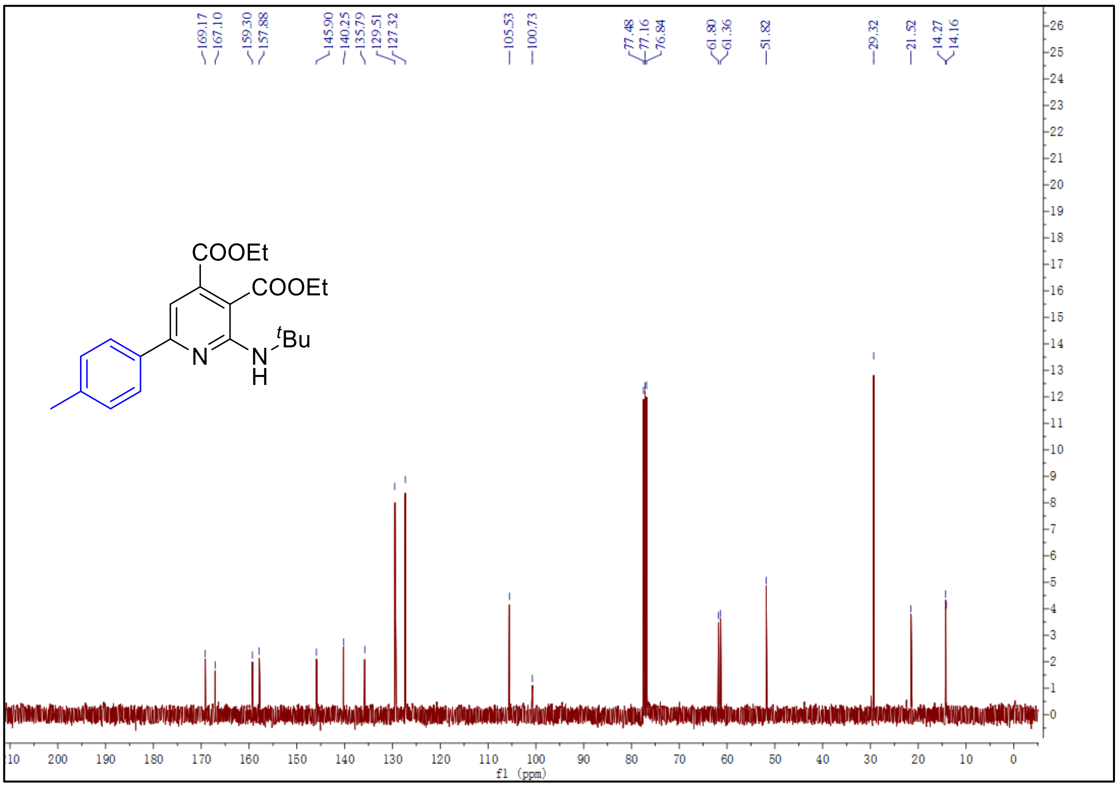

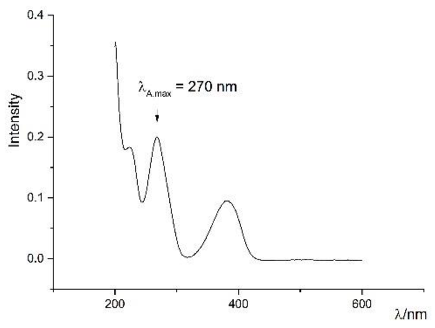

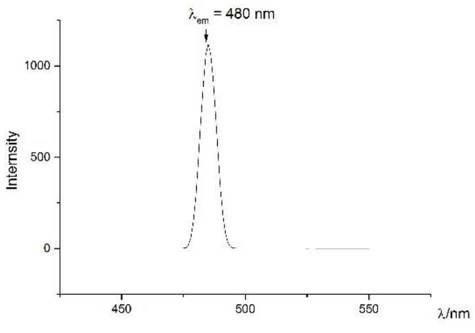

Figure S6. Diethyl 2-(*tert*-butylamino)-6-(o-tolyl)pyridine-3,4-dicarboxylate (**6**, $CDCl_3$)

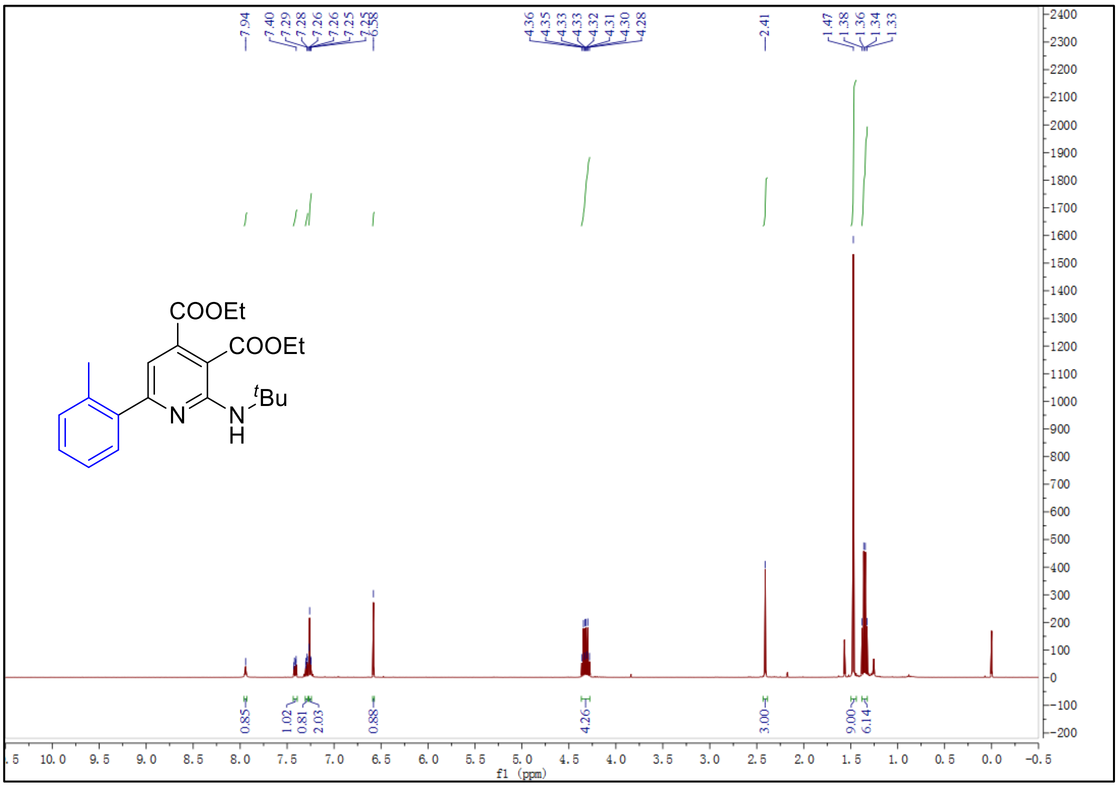

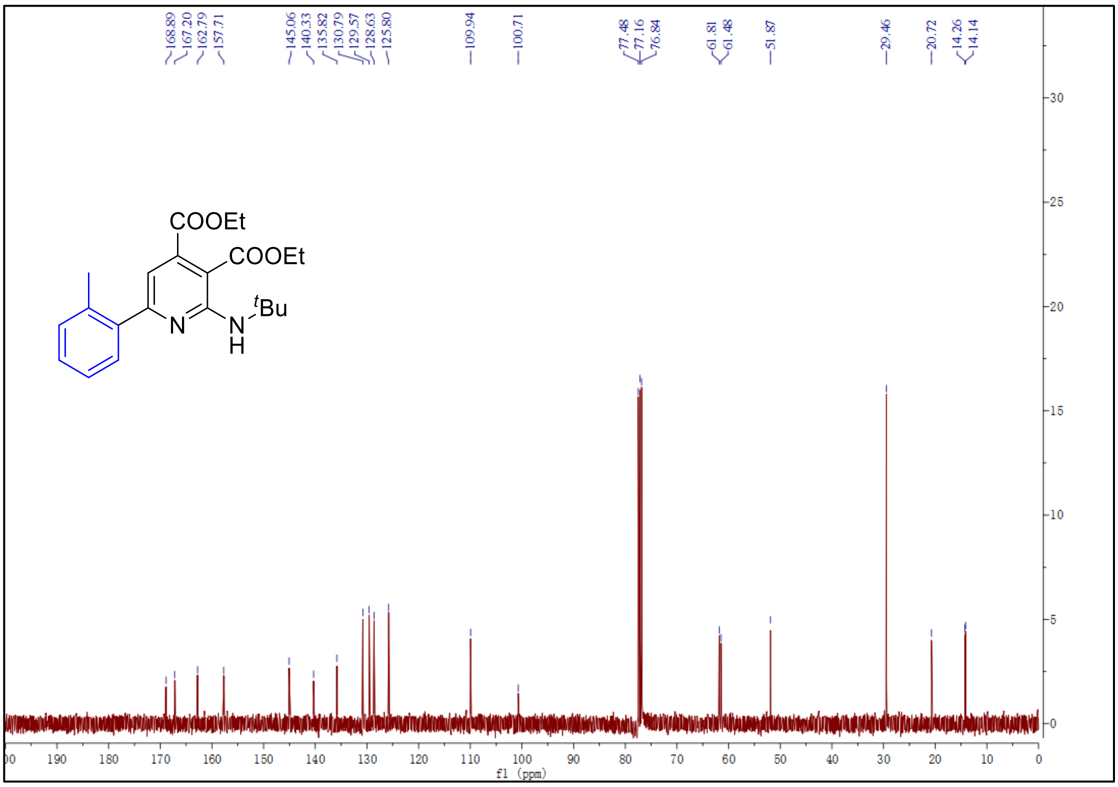

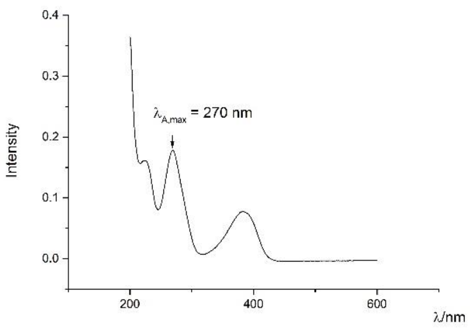

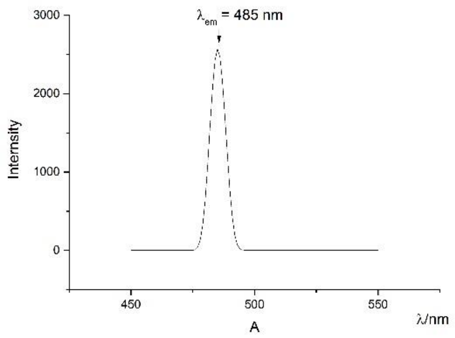

Figure S7. Diethyl 2-(*tert*-butylamino)-6-(4-bromophenyl)pyridine-3,4-dicarboxylate (**7**, $CDCl_3$)

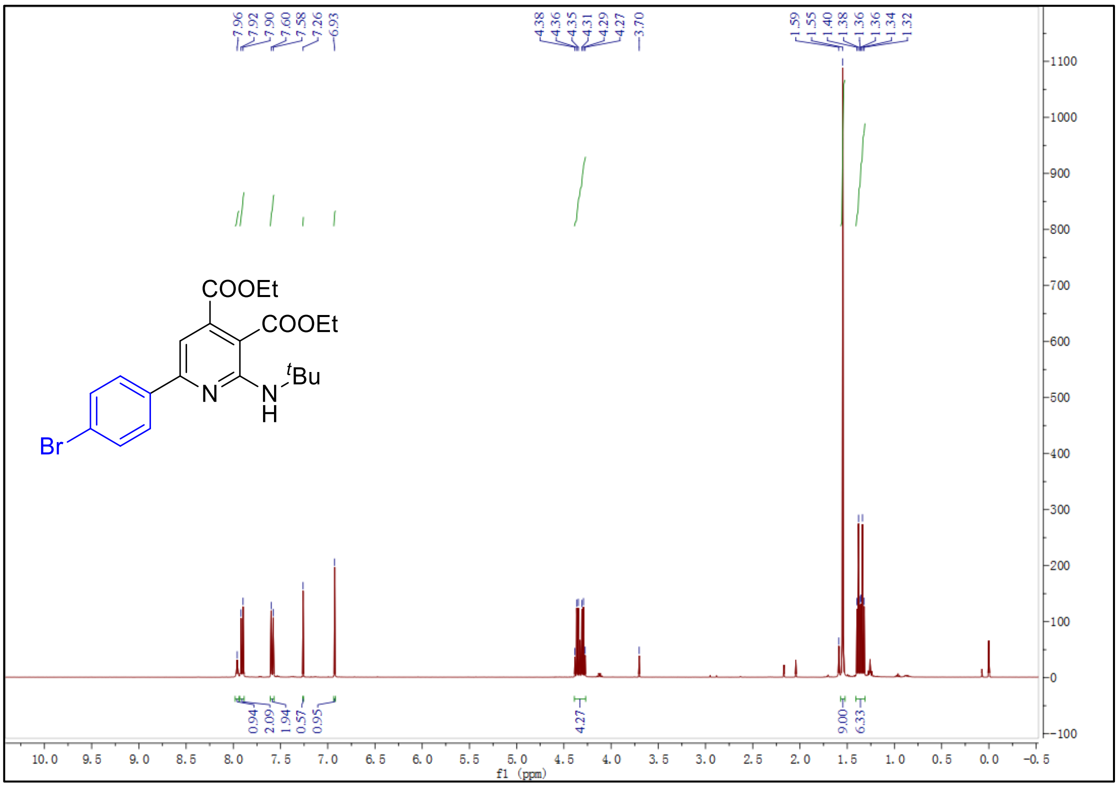

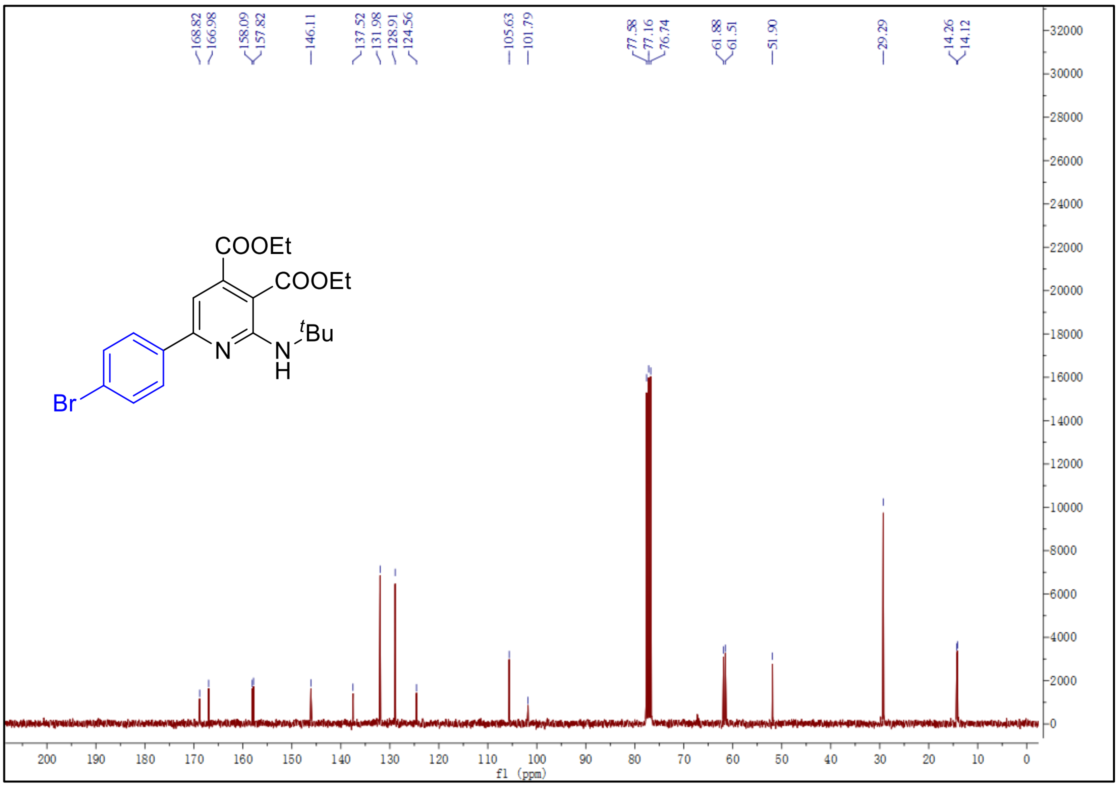

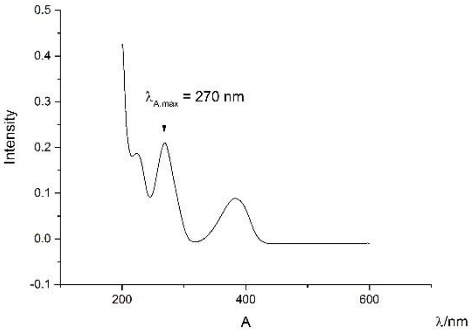

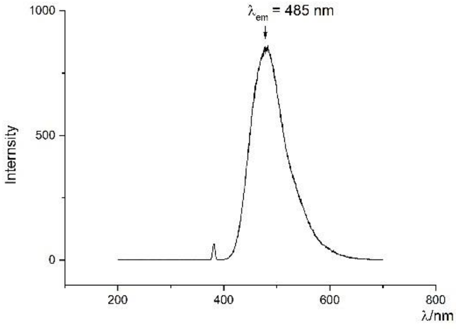

Figure S8. Diethyl 2-(*tert*-butylamino)-6-octylpyridine-3,4-dicarboxylate (**8**, $CDCl_3$)

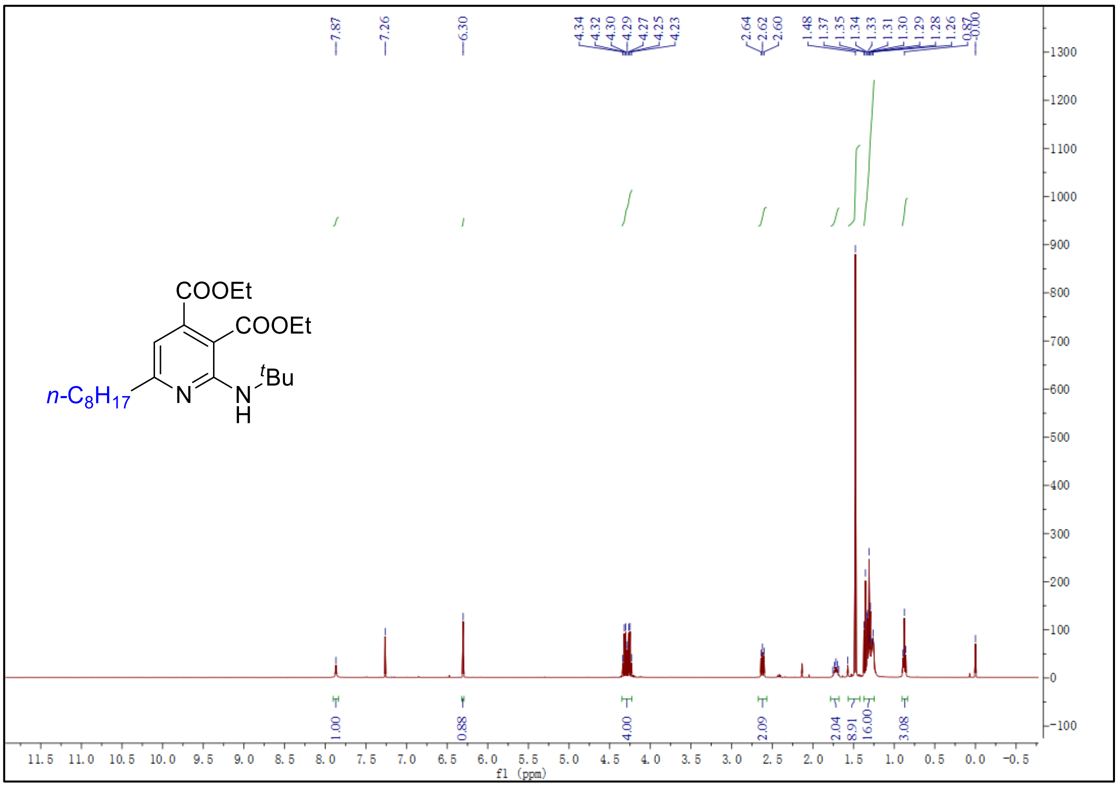

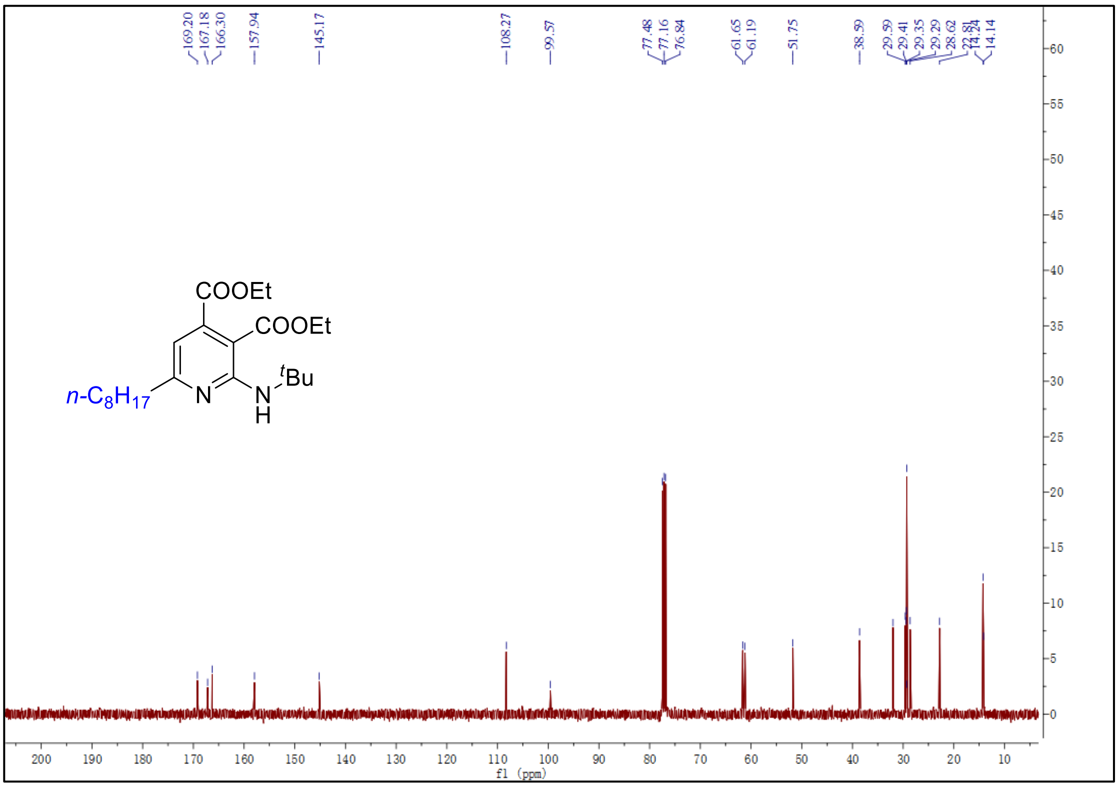

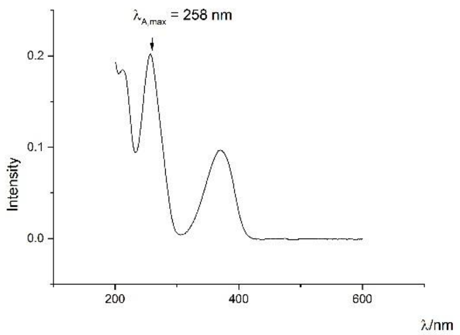

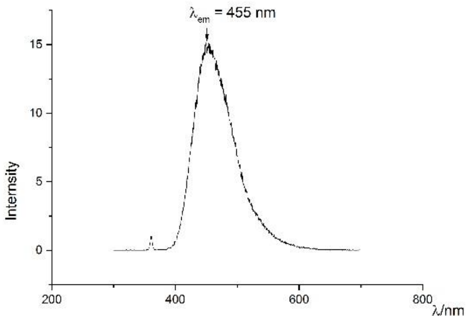

Figure S9. Dimethyl 2-(cyclohexylamino)-6-phenylpyridine-3,4-dicarboxylate (**9**, $CDCl_3$)

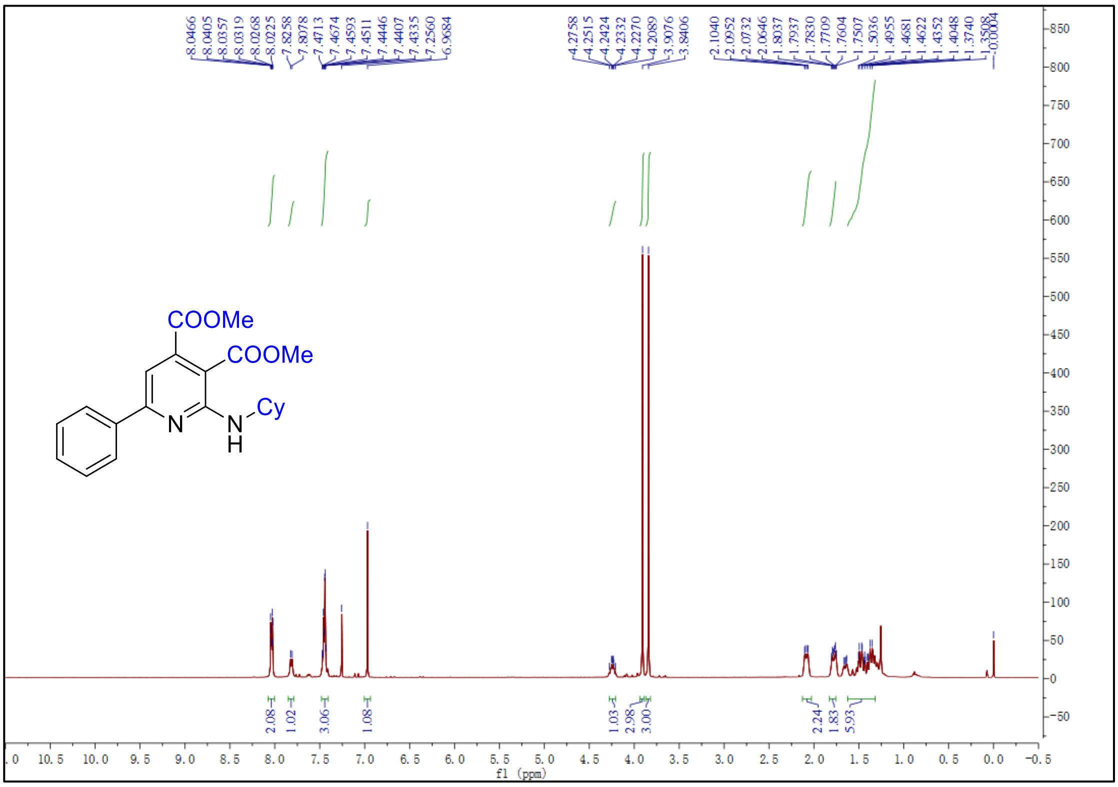

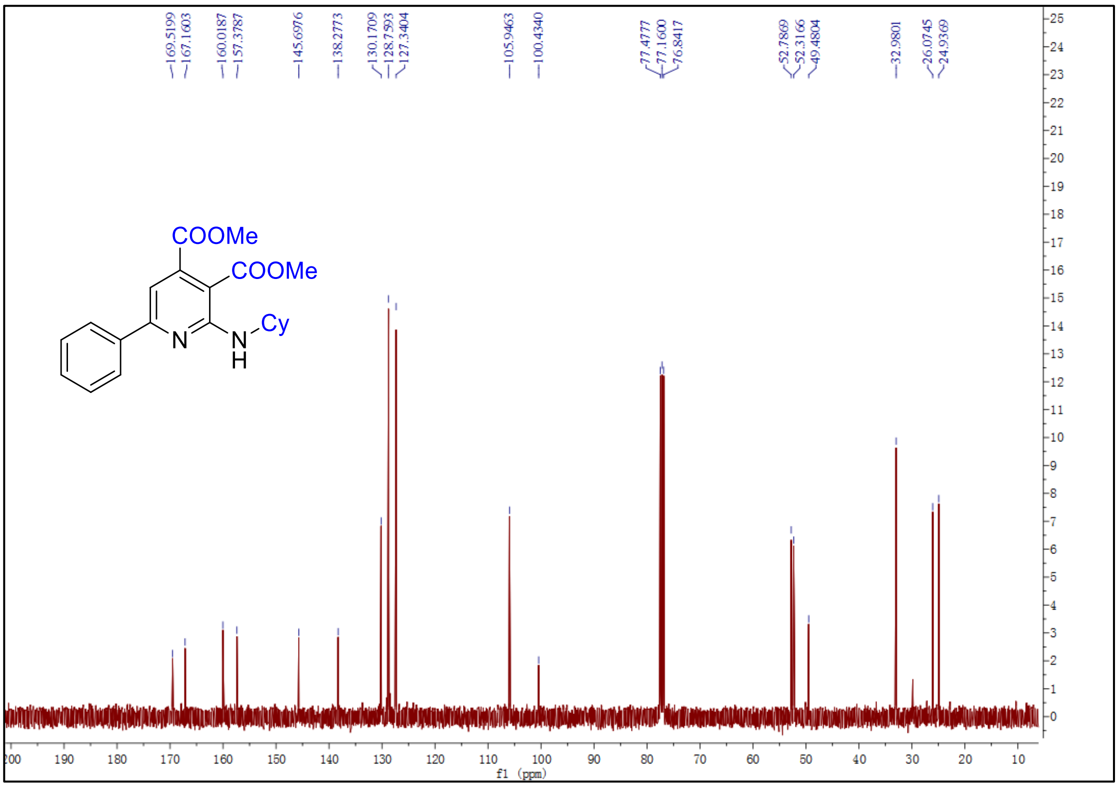

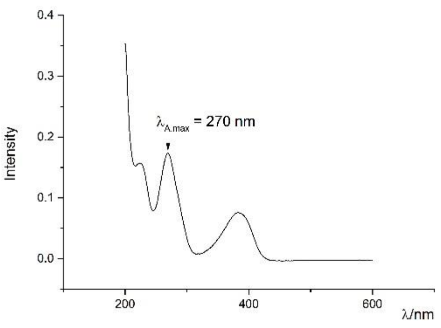

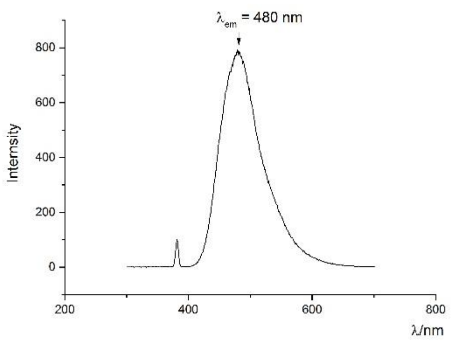

Figure S10. Bis(2-methoxyethyl) 2-(*tert*-butylamino)-6-phenylpyridine-3,4-dicarboxylate (**10**, $CDCl_3$)

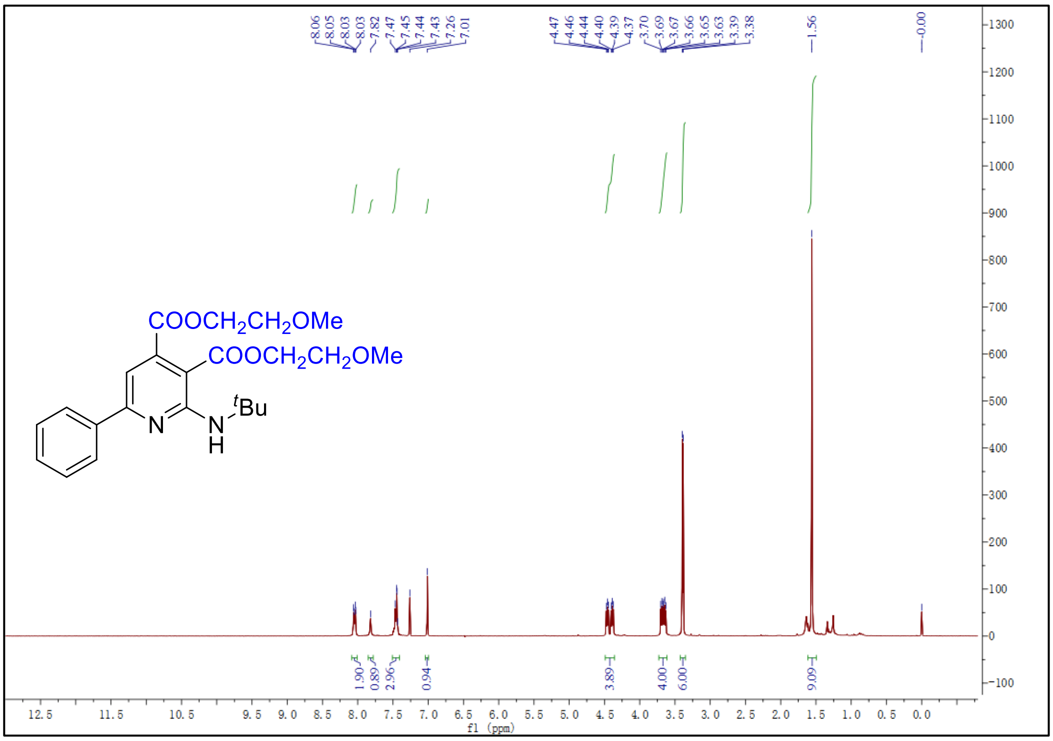

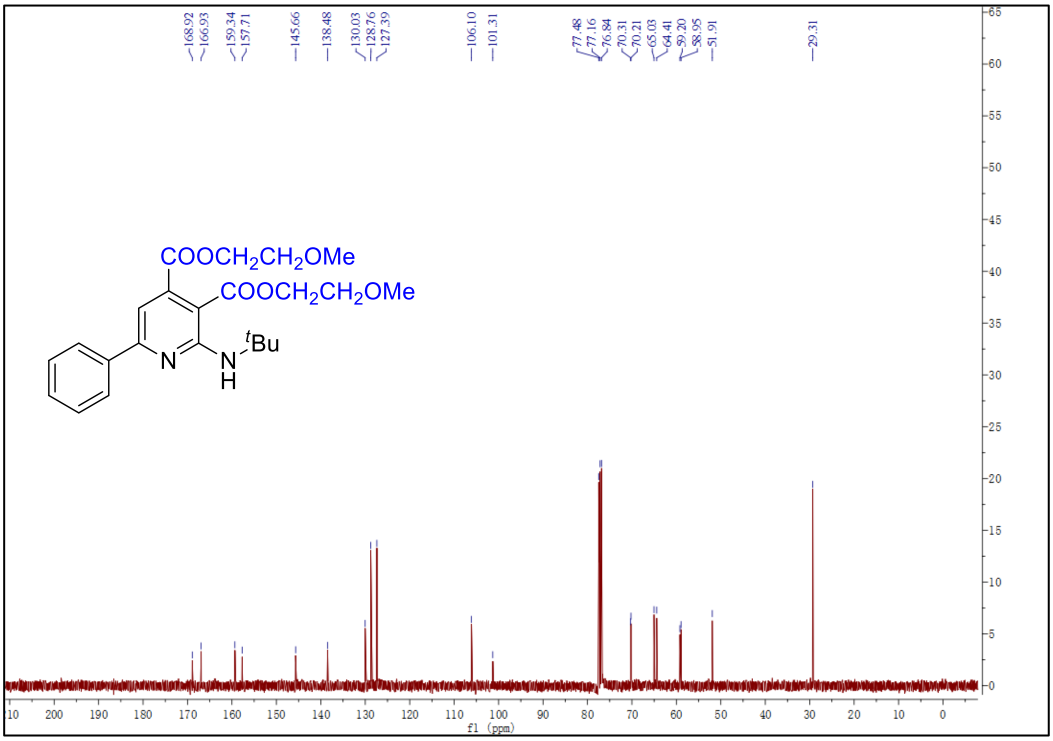

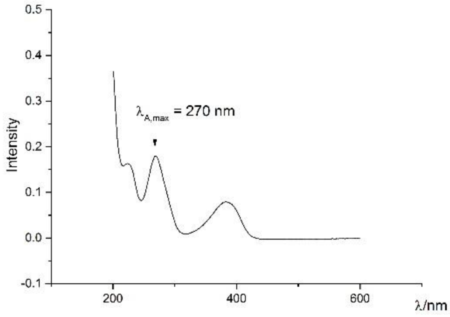

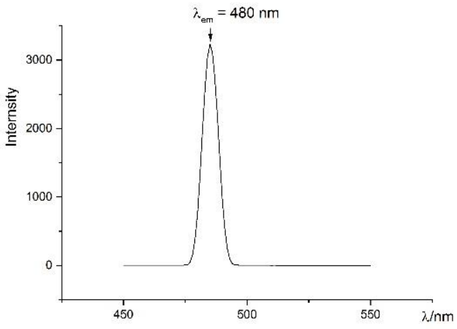

Figure S11. Diethyl 2-amino-6-phenylpyridine-3,4-dicarboxylate (**11**, $CDCl_3$)

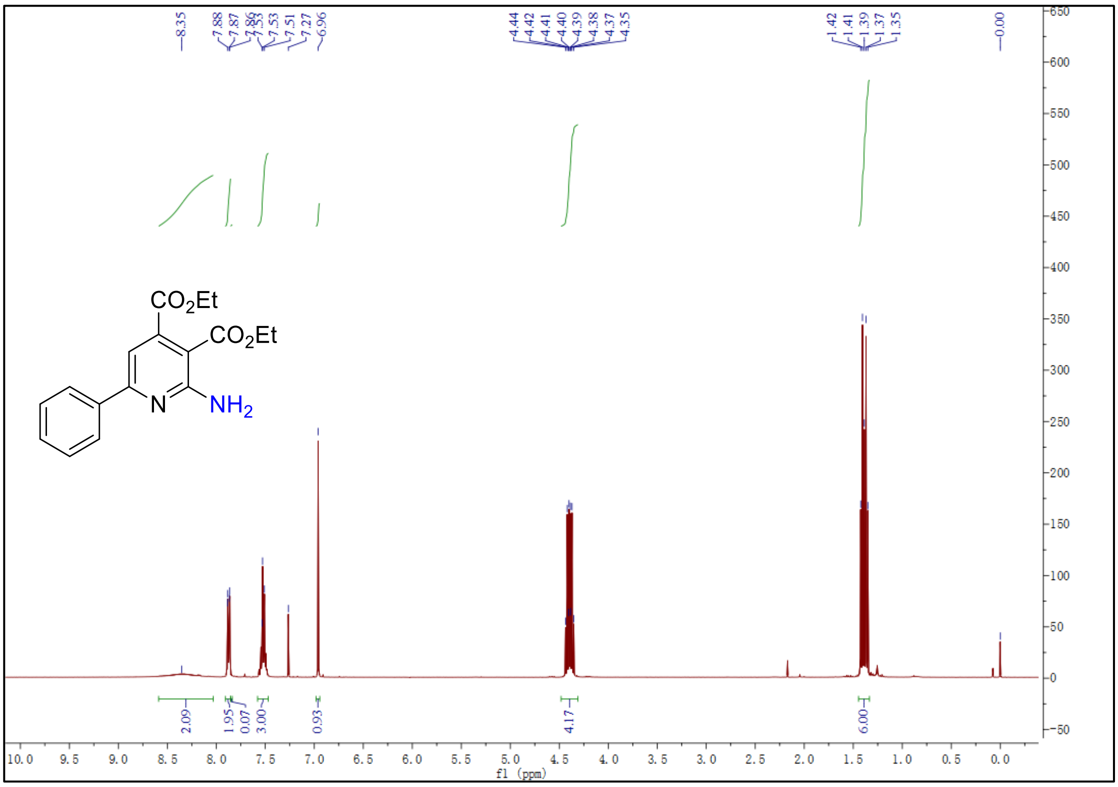

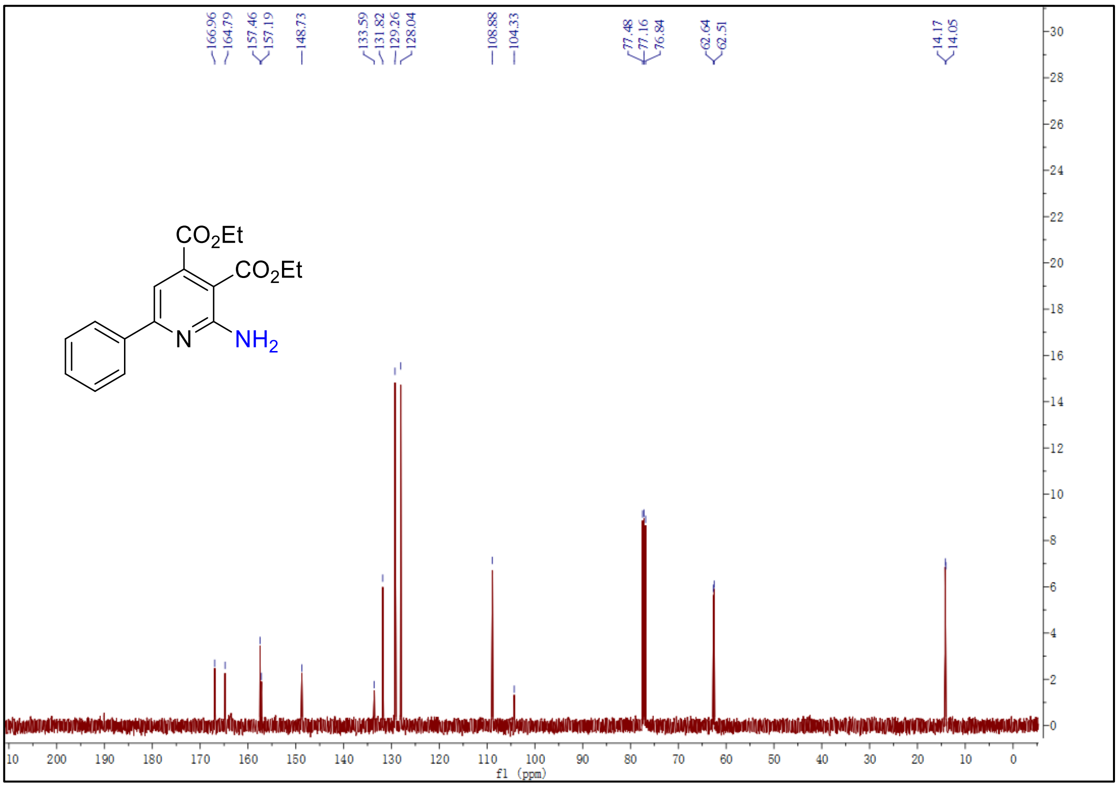

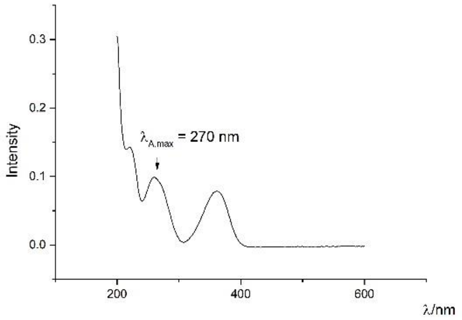

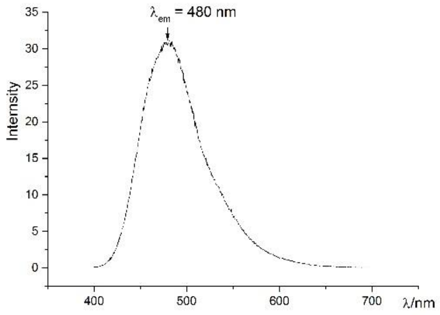

Figure S12. 2-(*tert*-Butylamino)-6-phenylpyridine-3,4-dicarboxylic acid (**12**, DMSO-*d6*)

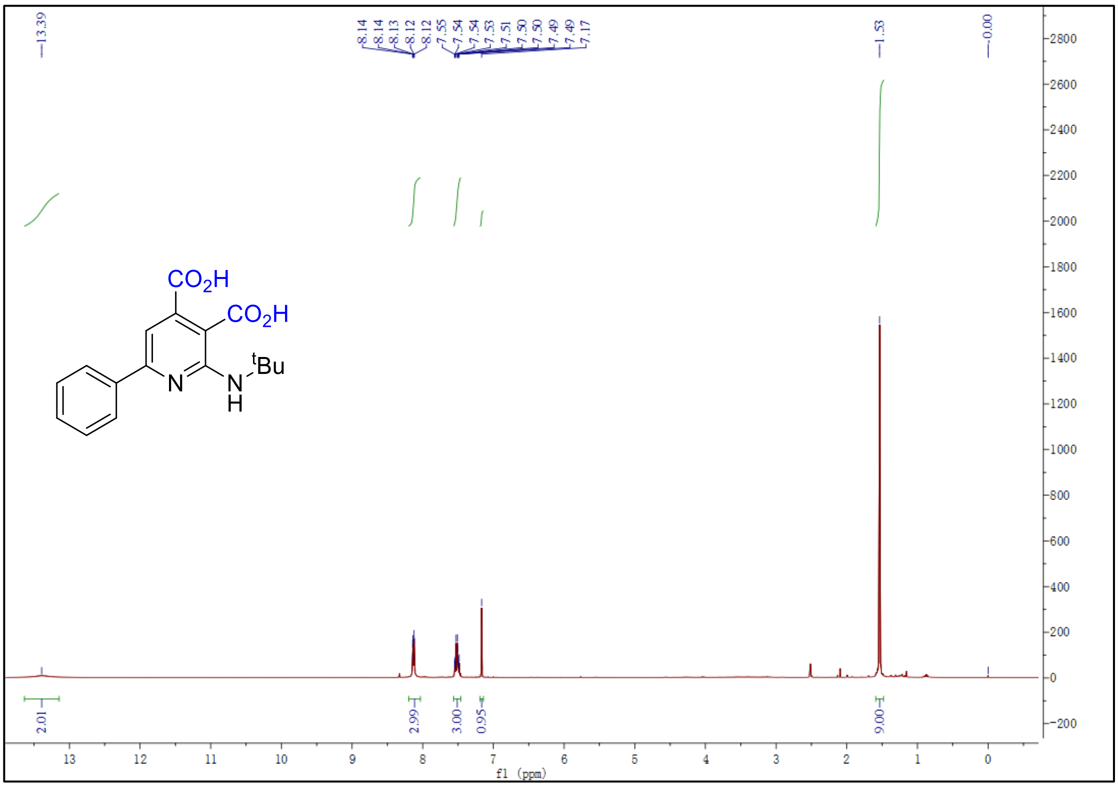

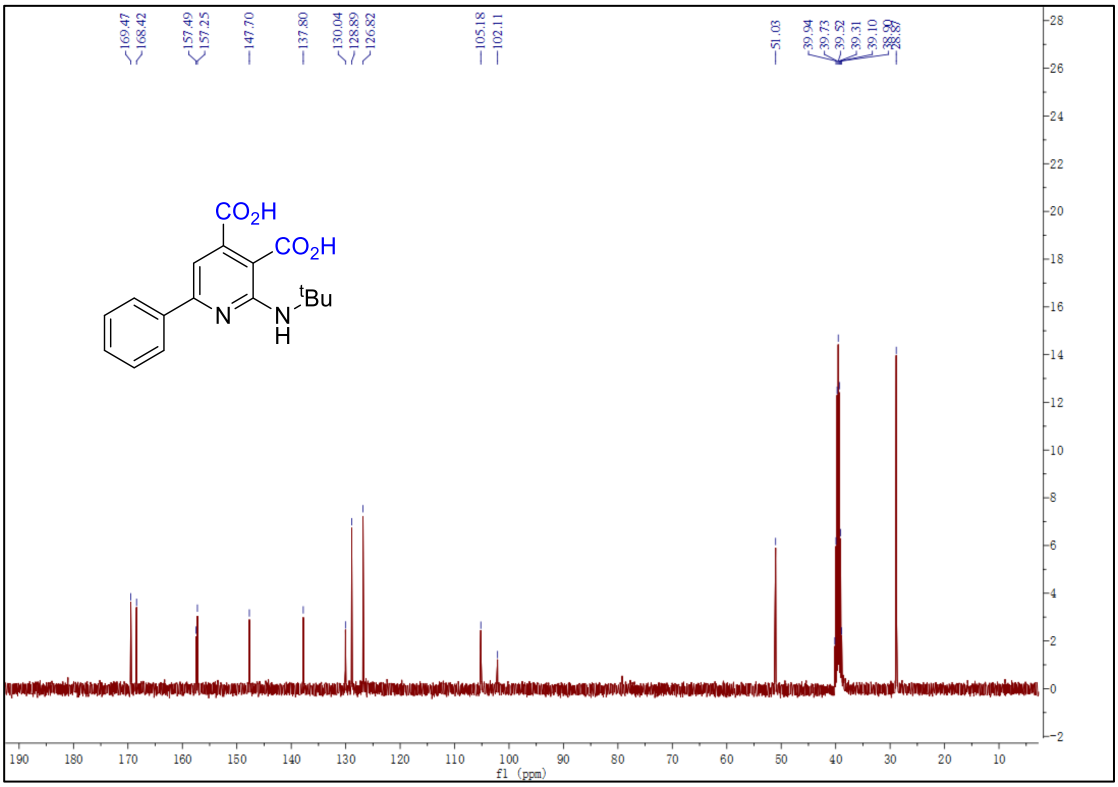

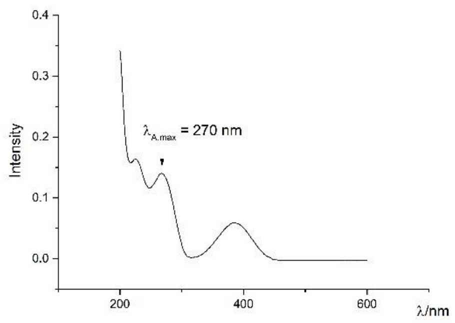

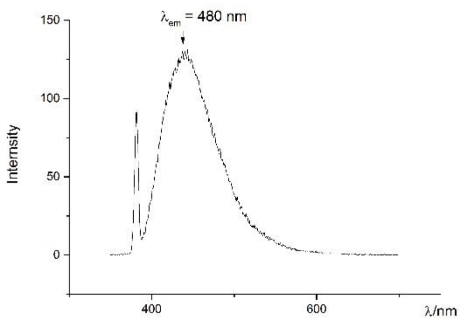

Figure S13. (2-(*tert*-Butylamino)-6-phenylpyridine-3,4-diyl) dimethanol (**13**, $CDCl_3$)

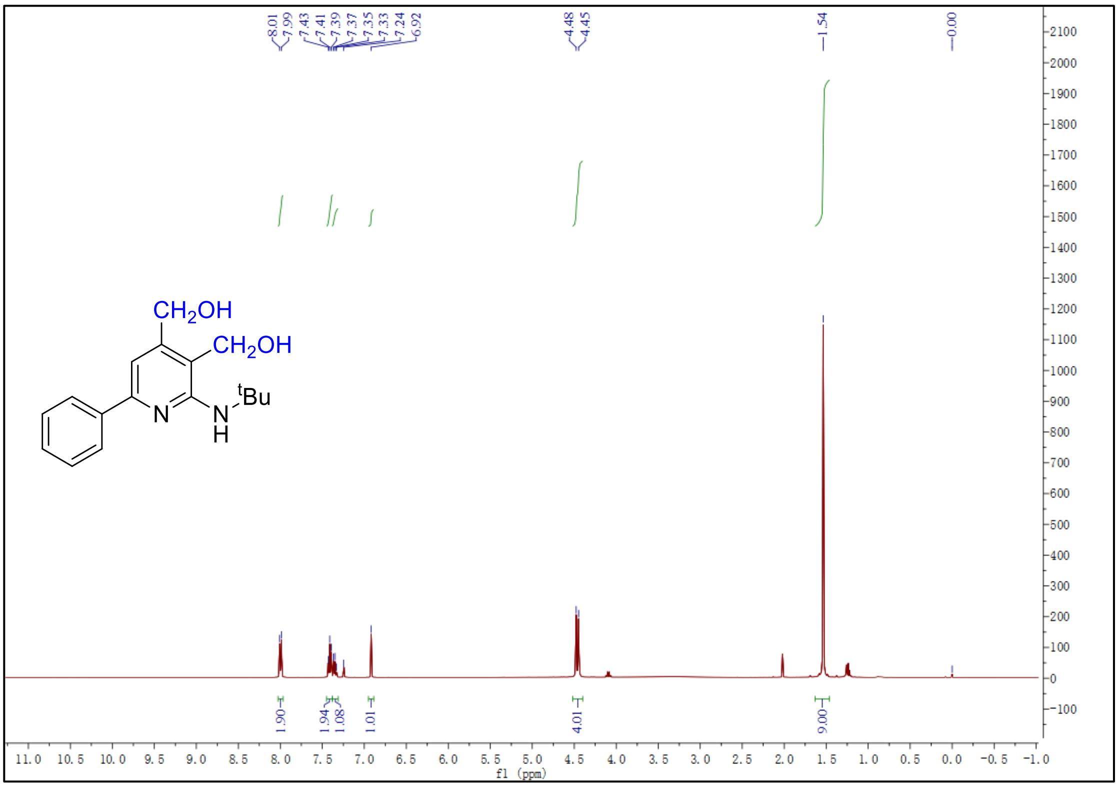

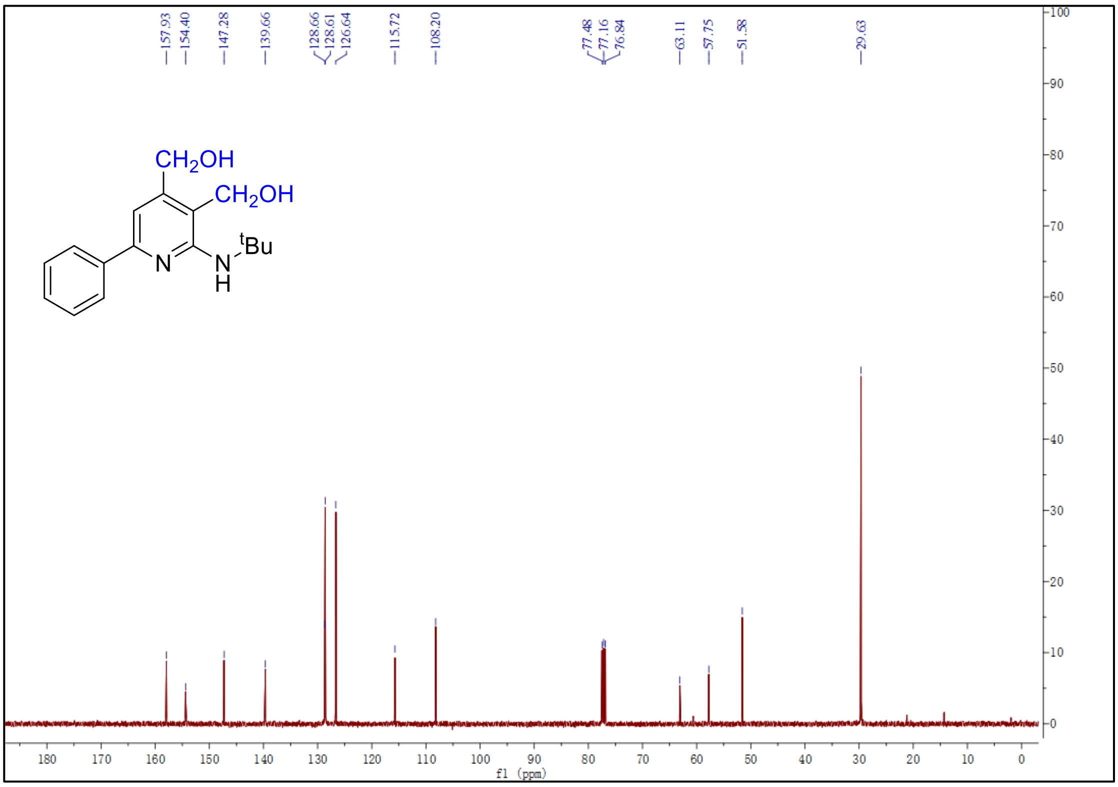

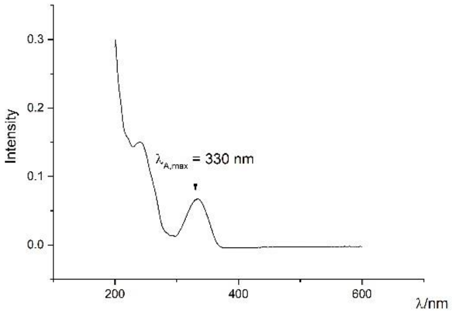

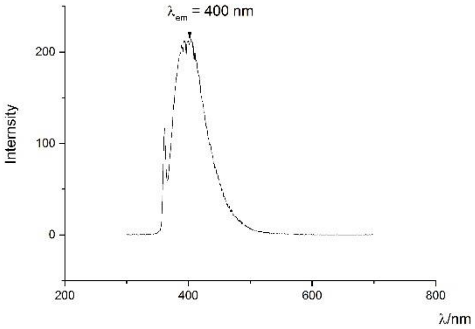

Figure S14. Diethyl 6-(4-((*tert*-butoxycarbonyl)amino)phenyl)-2-(cyclohexylamino)pyridine-3,4-dicarboxylate (**14**, $CDCl_3$)

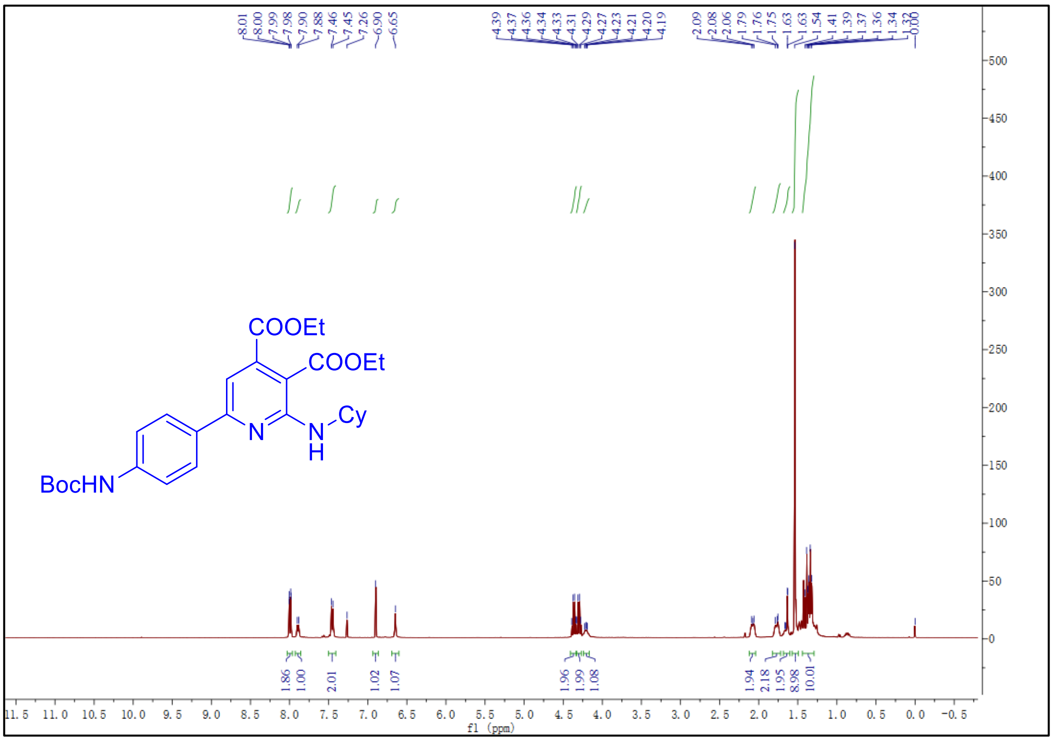

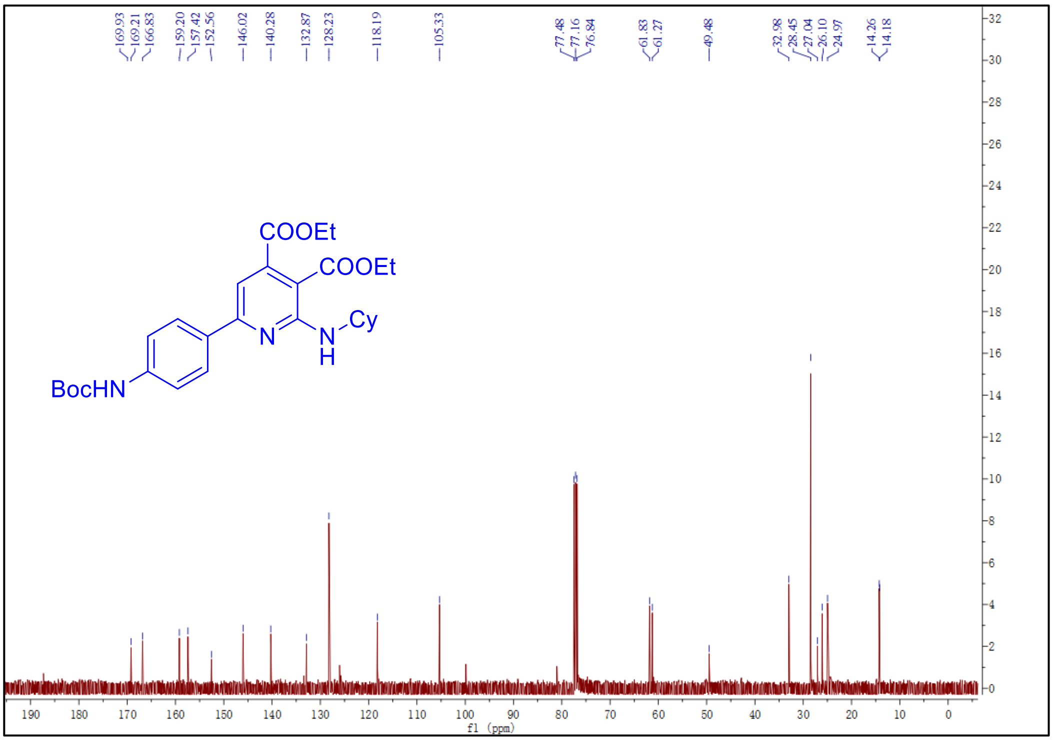

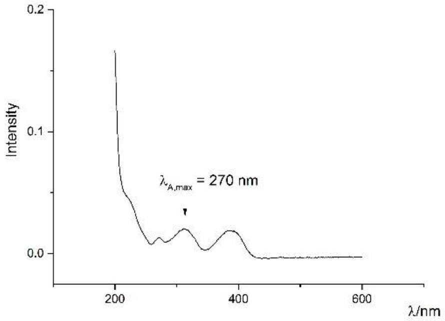

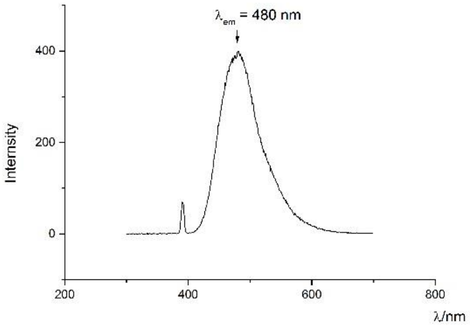

Figure S15. Diethyl 6-(4-azidophenyl)-2-(cyclohexylamino)pyridine-3,4-dicarboxylate (**15**, $CDCl_3$)

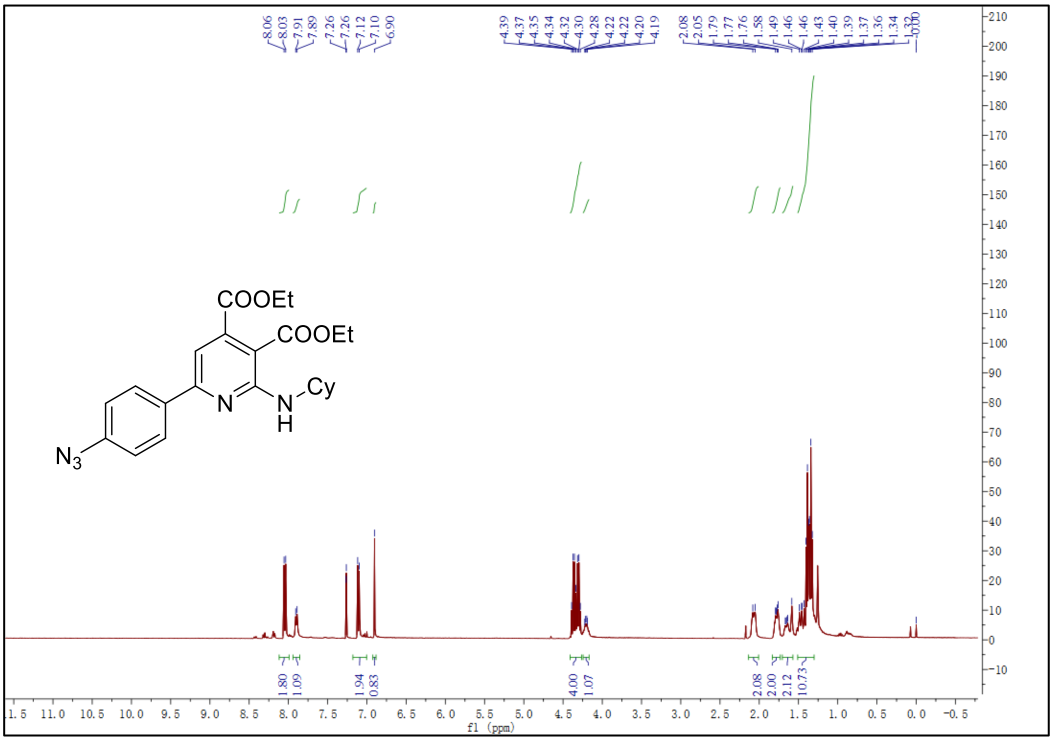

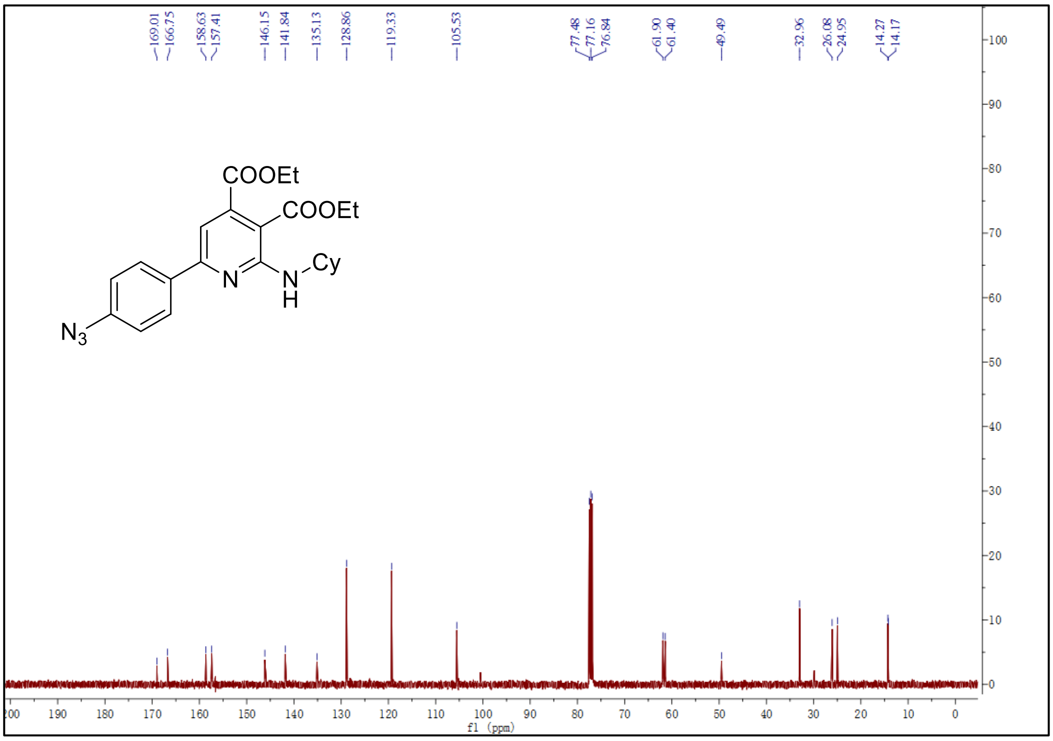

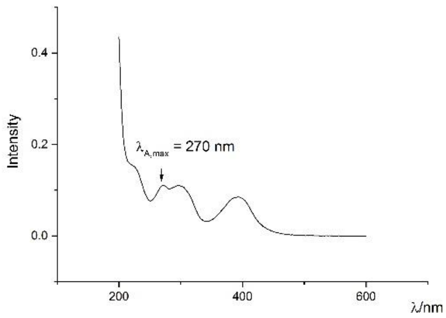

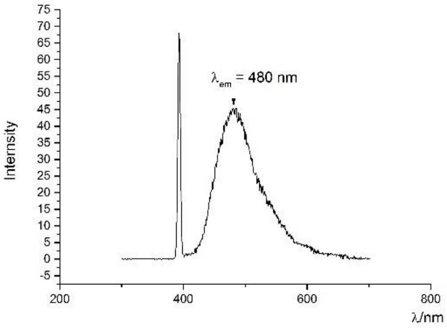

Figure S16. Diethyl 2-(cyclohexylamino)-6-(4-(4-phenyl-1*H*-1,2,3-triazol-1-yl)phenyl)pyridine-3,4-dicarboxylate (**16**, $CDCl_3$)

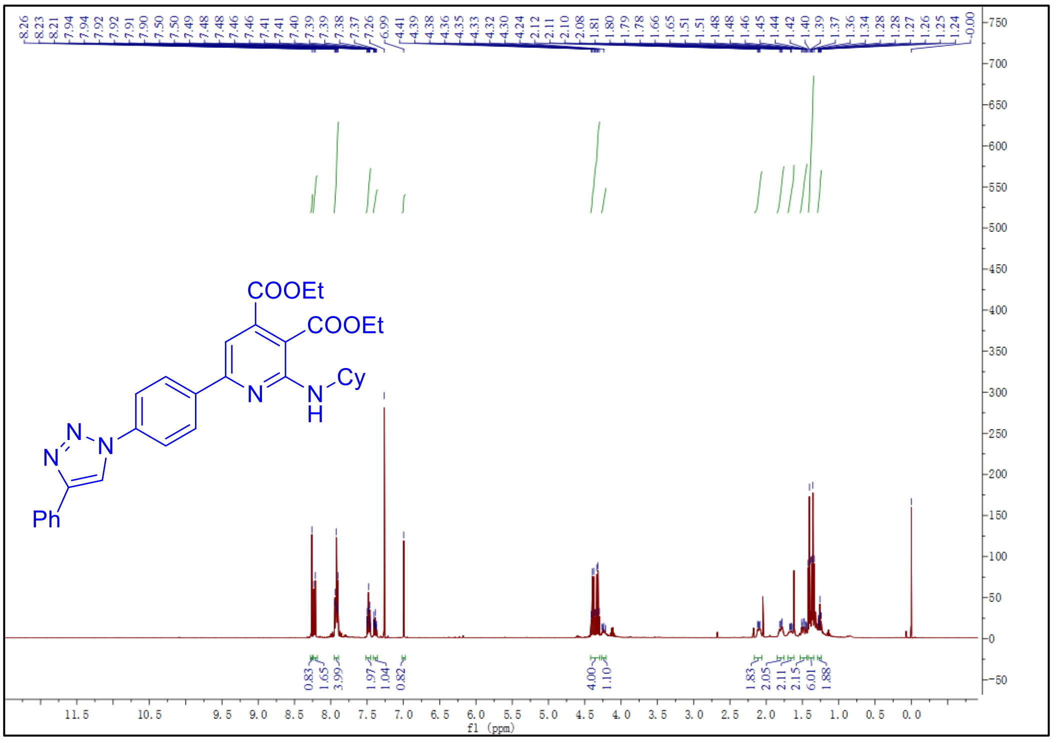

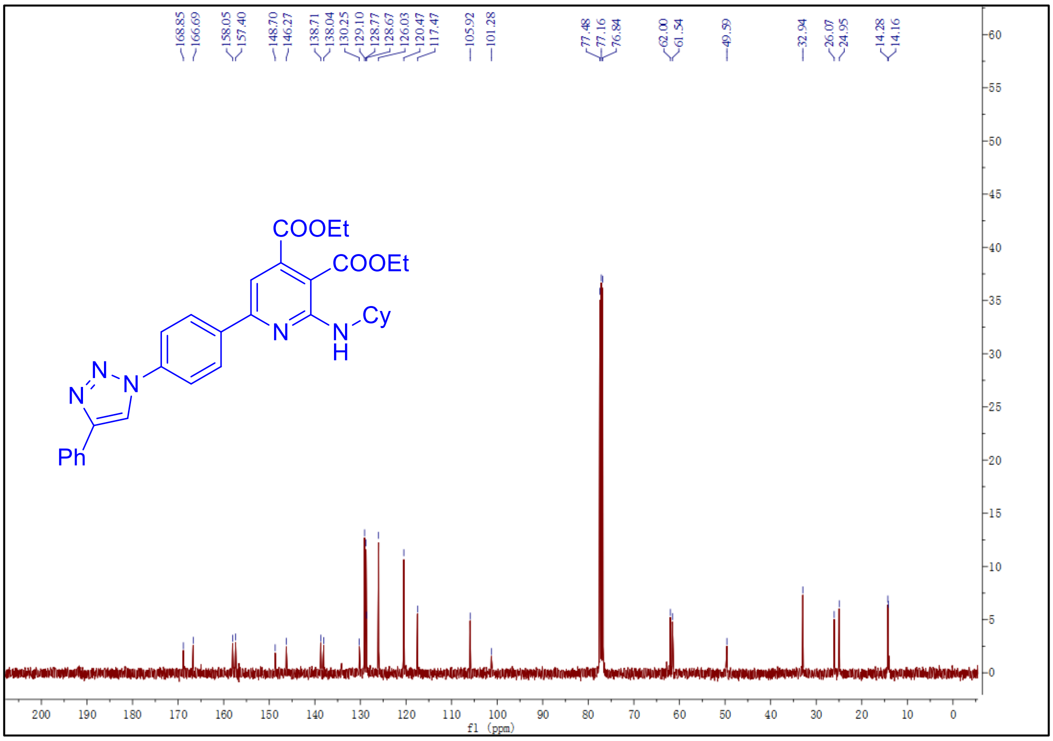

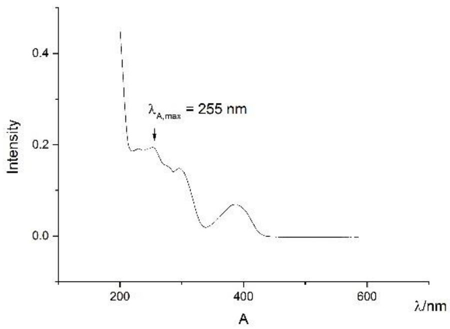

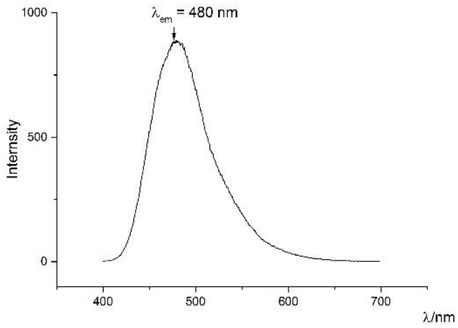

Figure S17. Diethyl 2-(cyclohexylamino)-6-(4-(4-(hydroxymethyl)-1*H*-1,2,3-triazol-1-yl)phenyl)pyridine-3,4-dicarboxylate (**17**, $CDCl_3$)

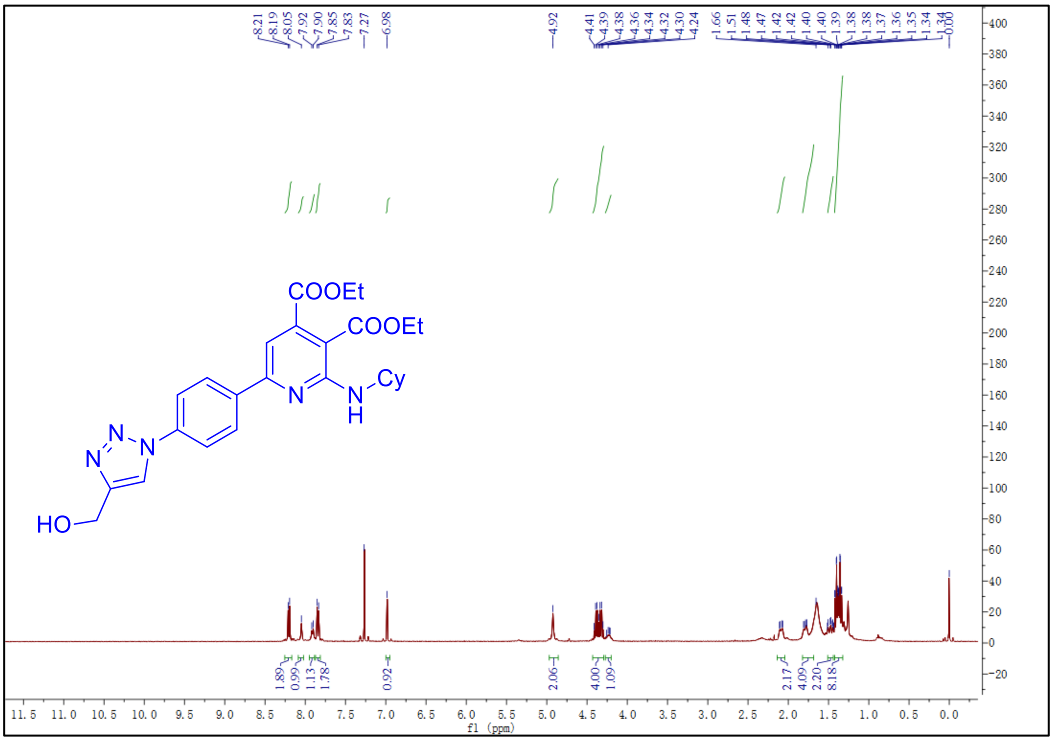

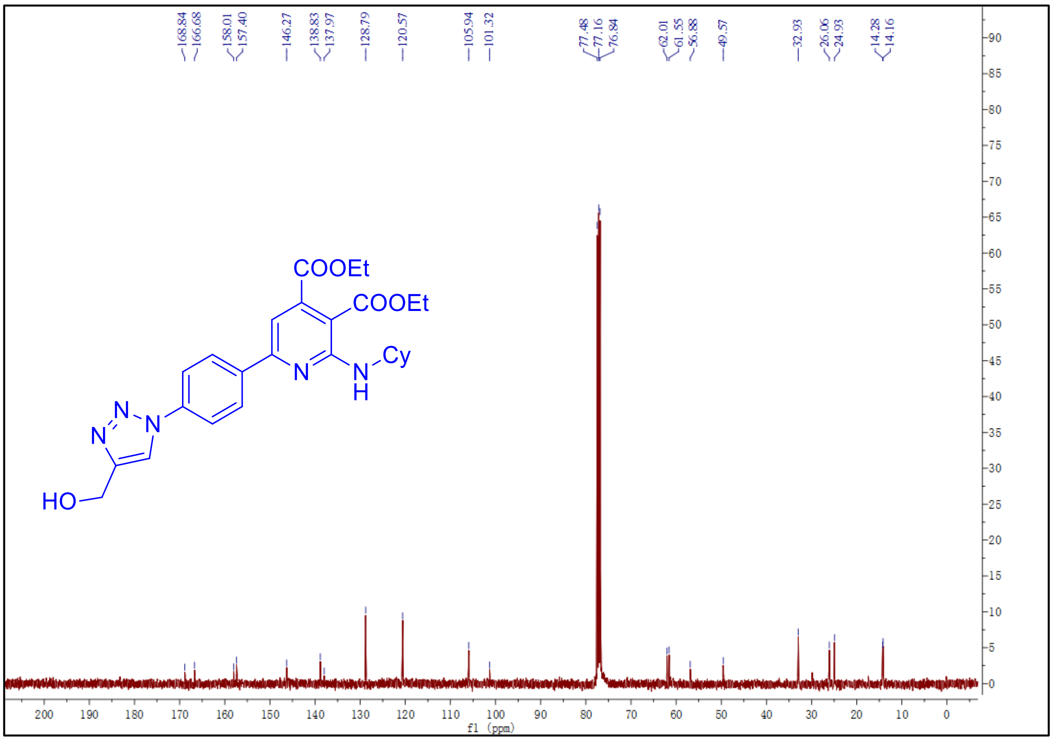

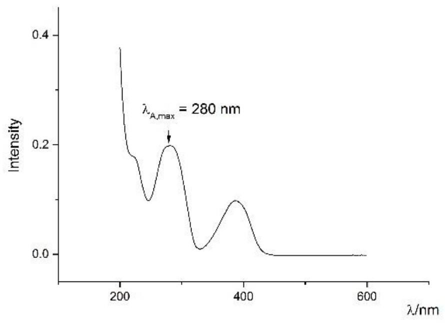

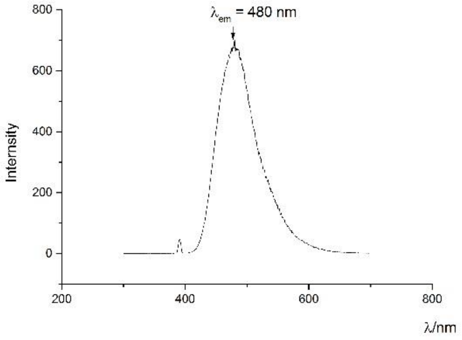

Figure S18. Fluorescence scan of SDS-PAGE gel of BSA protein by compound **15** and coomassie blue staining of SDS-PAGE gel of BSA protein by compound **15**

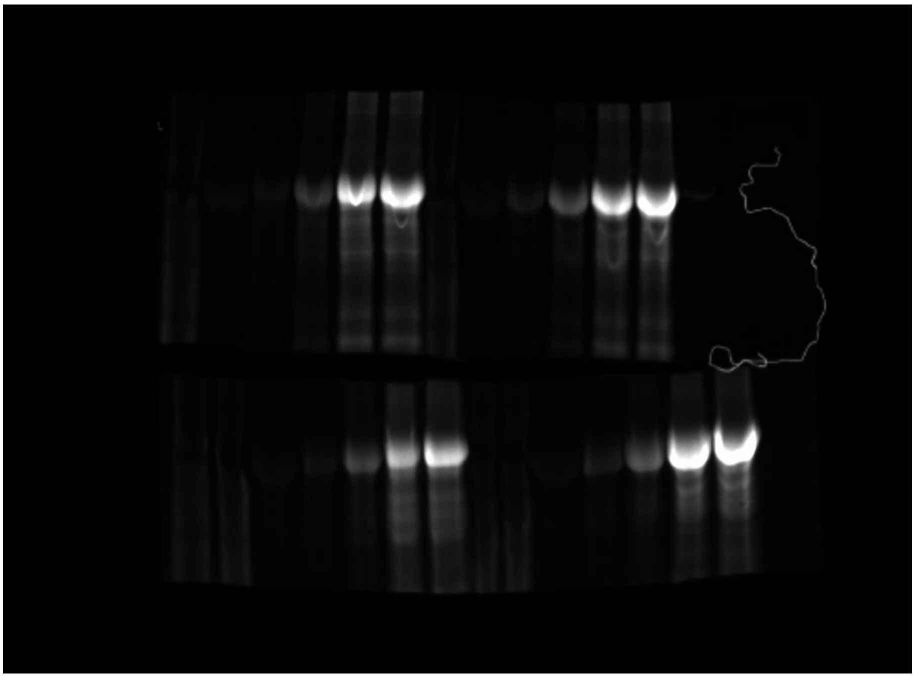

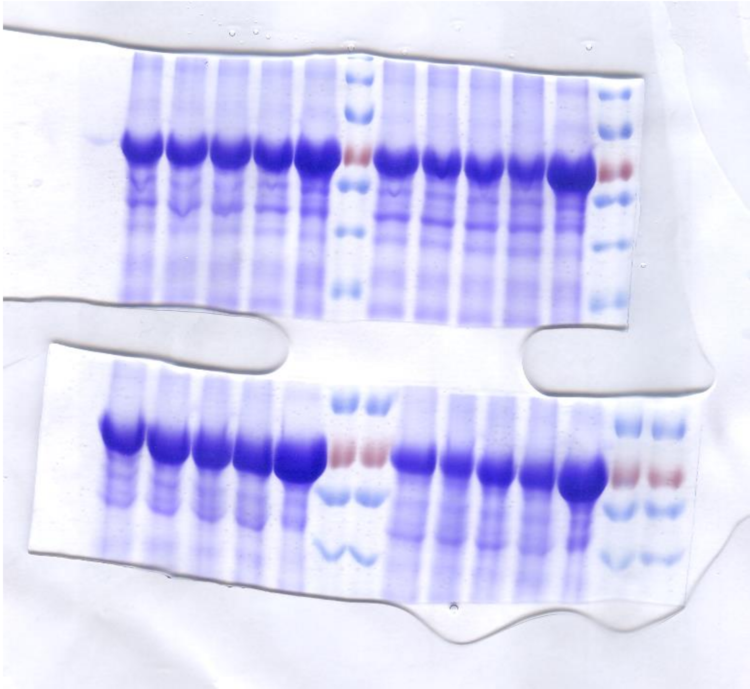

Supplement: Supplementary file 1 [file molecules-27-01596-s001.zip › molecules-1579221-supplementary.pdf]
